# Volatile Oil Profile of Prickly Ash (*Zanthoxylum*) Pericarps from Different Locations in China

**DOI:** 10.3390/foods10102386

**Published:** 2021-10-08

**Authors:** Yao Ma, Jieyun Tian, Yabing Chen, Ming Chen, Yulin Liu, Anzhi Wei

**Affiliations:** 1 College of Forestry, Northwest A&F University, Xianyang 712100, China; mayao277000@nwafu.edu.cn (Y.M.); tianjieyun@nwafu.edu.cn (J.T.); 2019055403@nwafu.edu.cn (Y.C.); 15511031817@nwafu.edu.cn (M.C.); liuyulin@nwafu.edu.cn (Y.L.); 2 Research Centre for Engineering and Technology of Zanthoxylum, State Forestry Administration, Xianyang 712100, China

**Keywords:** volatile oil profile, gas chromatograph-mass spectrometer, geographical variation, environmental factors, chemometric analyses

## Abstract

Volatile oils of prickly ash (*Zanthoxylum*) pericarps have various potential biological functions with considerable relevance to food, pharmacological, and industrial applications. The volatile profile of oils extracted from prickly ash pericarps obtained from 72 plantations in China was determined by gas chromatography and mass spectrometry. Several chemometric analyses were used to better understand the volatile oil profile differences among different pericarps and to determine the key factors that affected geographical variations in the main volatile constituents of oils. A total of 47 constituents were detected with D-limonene, alfa-myrcene, and linalool as the most abundant. The volatile profile of pericarp oils was significantly affected by prickly ash species and some environmental factors, and the key factors that affected volatile profile variations for different prickly ash species were diverse. Chemometric analyses based on the volatile oil profile could properly distinguish *Z. armatum* pericarps from other pericarps. This study provides comprehensive information on the volatile oil profile of pericarps from different prickly ash species and different plantations, and it can be beneficial to a system for evaluating of pericarp quality. Moreover, this study speculates on the key environmental factors that cause volatile oil variations for each species, and can help to obtain better prickly ash pericarp volatile oils by improving the cultivated environments.

## 1. Introduction

Prickly ash (*Zanthoxylum*) comprises approximately 250 known species of perennial trees and shrubs [1], and the trees of these species are distributed across a wide range of regions due to their high resistance to adverse climate and soil conditions and huge economic gains [2]. Because of the difference in tree species, climate, soil conditions, and management measures, prickly ash pericarps vary in morphology and active constituents resulting in differences in quality, price, and therapeutic efficacy [3,4,5]. The common species which are widely cultivated and utilized in China are *Z. bungeanum* Maxim. (ZB) and *Z. armatum* DC (ZA) [4,6,7]. Dry ZB pericarps, named Pericarpium Zanthoxyli (‘Huajiao’), are listed in the Pharmacopoeia of the People’s Republic of China as an important medicine to treat diseases [8]. The plants of this species are classified into two groups: the first group (*Z. bungeanum* 1, ZB1) is represented by samples from Hancheng and the second group (*Z. bungeanum* 2, ZB2) is represented by samples from Fengxian [9,10]. The plants of ZA are mainly distributed in southern China [4,7], and ZA pericarps are popular in Sichuan cuisine, especially in hotpot. The pericarps of ZA and ZB are commonly used as spices for their special flavor and are a therapeutically efficacious traditional medicine [11,12]. The therapeutic efficacy of the two popular species is mainly because of the presence of volatile oils in their pericarps [13,14,15]. Further, volatile oils contribute to the pungent flavor of the pericarps [4]. 

Volatile oils are synthesized by organs in aromatic plants as defense and reproductive mechanisms [16], and they are a complex blend of substances (from a dozen to several hundred constituents) that are present at different concentrations [17,18]. Generally, two or three major constituents are present in high concentrations (>30%), while other constituents are present only at trace levels [19]. Monoterpenes, sesquiterpenes, and their oxygenated derivatives are the most common constituents, but some trace constituents are also important in some volatile oils [20]. Generally, the volatile oil profile varies with inter- and intra-specific variation, climate and soil conditions, maturity and harvest time, and storage and extraction methods [21,22,23]. Prickly ash trees are widely cultivated in China with diverse environmental conditions, and some species overlap in the same producing areas. The discrimination of species and origins is difficult due to the numerous species, incongruence in vernacular names, and similarity in the morphology of some species and origins. The volatile oil profile coupled with chemometrics has been used as a biological strategy for the identification of the species and origin of some plants [16,20]. Hence, a comprehensive and comparative study on prickly ash pericarp volatile oil profiles from different species and different producing areas is required.

This study aims to determine the influence of prickly ash species and environmental factors on the pericarp volatile oil profile. The pericarps from 72 different plantations were collected, and the volatile oil profile of these pericarps was detected using gas chromatography and mass spectrometry (GC-MS). Several chemometric methods were conducted to better understand the volatile oil profile differences among different pericarps. Moreover, redundancy analysis (RDA) was used to better understand the relationship between the volatile oil profile and environmental factors (location, climate, and soil conditions). The proposed approach is a useful tool for determining the volatile oil profile differences of different prickly ash species and the key environmental factors that cause variations of the volatile oil profile in pericarps. Hence, this study will help to improve the volatile oil quality of pericarps by introducing better prickly ash species and improving the cultivated environments.

## 2. Materials and Methods

### 2.1. Sample Collection and Preparation

Prickly ash pericarps were obtained from 72 plantations across 12 provinces (Shandong, Hebei, Shanxi, Shaanxi, Henan, Gansu, Qinghai, Sichuan, Chongqing, Guizhou, Jiangxi, and Yunnan) in 2018. The ZA pericarp samples were from 17 plantations, the ZB1 pericarp samples were from 29 plantations, the ZB2 pericarp samples were from 13 plantations, and the other pericarp samples (derived from some relevant *Zanthoxylum* species) were from 13 plantations. About 0.5 kg of mixed topsoil samples (0–5 cm) were collected from five sites on each plantation, and 5.0 kg of mixed fruit samples were collected from five random trees on each plantation. The impurities of the topsoils and fruit samples were removed, and three biological repetitions were created for each plantation for testing the fruit and topsoil samples. Location information on the samples was recorded (Table S1), and collected samples in valve bags from each plantation were transported to the laboratory. The pericarps were separated from the dried fruit samples, and then the dried soil and pericarp samples were each ground to a homogenized powder.

### 2.2. Determination of Environmental Factors

The location information for the plantations, that is, the sampled longitude (Long), latitude (Lat), and altitude (Alt), was obtained from a GPS real-time altitude app developed by Fuzhou Lexun Network Technology Co., LTD (Fuzhou, China); the climate information for the plantations, that is, the mean atmospheric pressure (AtP), mean temperature (MT), mean relative humidity (MRH), and mean annual precipitation (MAP), was collected from the National Meteorological Information Centre (http://data.cma.cn/ accessed on 24 December 2018). For the determination of soil conditions, soil organic matter (OM) was detected using an external heating method with potassium dichromate concentrated sulfuric acid; the pH values in the soils (pH) were detected using a pH meter (Sartorius AG, Goettingen, Germany) in a soil: water suspension (1:2.5; v:v); the total nitrogen content in the soils (N_t_) was detected using an AA3 Auto Analyzer (Seal Analytical GmbH, Norderstedt, Germany); the available nitrogen content in the soils (N_a_) was detected using the alkaline hydrolysis diffusion method; the total phosphorus content (P_t_) and available phosphorus content in the soils (P_a_) were detected using the molybdenum–antimony colorimetric method; the total potassium content (K_t_) and available potassium contents in the soils (K_a_) were detected using flame photometry (Shanghai Precision Science Instrument Co., Ltd. Shanghai, China); the content of aluminum (Al), cadmium (Cd), lead (Pb), manganese (Mn), and nickel (Ni) in the soil samples was detected using inductively coupled plasma optical emission spectrometry (PerkinElmer Co., Waltham, MA, USA); and the arsenic content in the soils (As) was detected using an AFS-2100 atomic fluorescence spectrophotometer (Beijing Haiguang Instrument Co. Ltd., Beijing, China) after the digestion of mixed acids. The detailed date of location, climate, and soil has been reported in our previous articles [9,24,25,26].

### 2.3. Qualitative and Quantitive Determinations of Prickly Ash Pericarp Volatile oil Profile

The extraction of volatile oils was conducted for 4 h using an all-glass Clevenger-type apparatus [17,19]. Dry pericarp powder samples (30.0 g, each) were soaked in distilled water (300 mL). The volatile oil content was expressed as the volume of 100 g of dry powder (mL/100 g). Measurements were collected in three replications. The collected oils were dried using anhydrous sodium sulphate and kept at 4 °C. Determination of the volatile oil profile in prickly ash pericarps was performed using GC-MS [17]. The GC-MS analysis was performed using the Thermo Scientific Trace 1310 gas chromatograph (Thermo Fisher Scientific Inc., Massachusetts, MA, USA) equipped with a flame ionization detector and a TG-5 MS column (30 m × 0.25 mm, 0.25 μm). 

Ten μL of diluted oils (1/1000; *v*/*v*, in n-hexane) were injected in the split mode. The MS transfer line and ion source temperature were both 280 °C. Helium gas with a purity of 99.99% was selected as the carrier gas at a flow rate of 30 mL/min. The oven temperature started at 35 °C for 3 min, and then the temperature gradually increased to 150 °C at a rate of 3 °C/min; the temperature then increased to 260 °C at a rate of 10 °C/min. For the last stage, the temperature increased to 290 °C at a rate of 5 °C/min, and it was maintained at 290 °C for 5 min. The electron impact ion source mode with an ionization energy of 70 eV, a scan time of 0.2 s, and mass range of 40–460 amu was used. The oil constituents were identified by comparing their mass spectra fragmentation with the National Institute of Standards and Technology (NIST) and by comparing the retention index (RI) with the results reported in the literature. Moreover, some main constituents were identified by comparing their mass spectra fragmentations with authentic standards (Shanghai Yuanye Bio-Technology Co., Ltd., Shanghai, China). The quantification of the main oil constituents based on peak area was carried out using the external standard method using the corresponding program (Table S2). The content of each oil constituent was expressed as milligrams of constituent per milliliter oil (mg/mL).

### 2.4. Data Analyses

Univariate statistical analysis was used to assess each variable independently with Tukey’s multiple comparisons (*p* < 0.05) in the IBM SPSS Statistics 20.0 software. The mean, median, standard deviation, variance, skewness coefficient of a variable, *p*-value for the Kolmogorov–Smirnov normality test, and their subsequently drawn histograms were used to assess whether the data came from a normally distributed population. The data were Z-scores transformed before chemometric analyses if the values did not conform to a normally distributed population (Tables S3–S5). A box plot, principal component analysis (PCA), and discriminant analysis (DA) were performed using OriginPro 2018C (Originlab, Northampton, USA). A cluster heat map (CHM) was performed using TBtools software. Orthogonal partial least squares discriminant analysis (OPLS-DA), t-distributed stochastic neighbor embedding (tSNE), and uniform manifold approximation and projection for dimension reduction (UMAP) were performed using a free online platform for data analysis (http://www.omicshare.com/tools accessed on 13 June 2021). Furthermore, RDA was performed using the Canoco 5.0 program.

## 3. Results

### 3.1. Volatile Oil Profile in Different Prickly Ash Pericarps

#### 3.1.1. Qualitative Determination of Volatile Oil Constituents in Prickly Ash Pericarps

**Figure 1:**
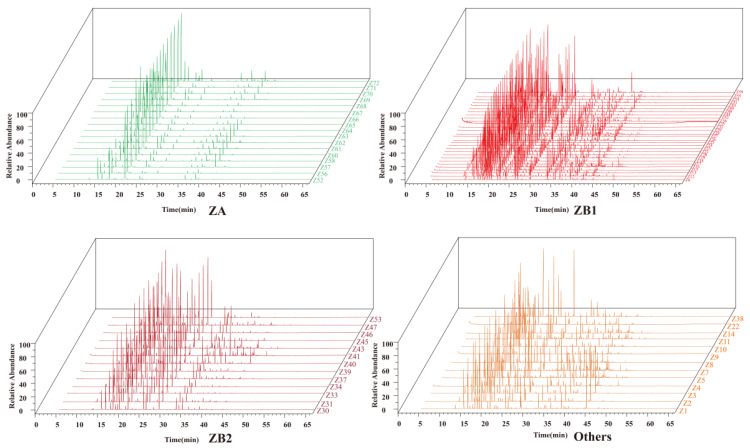
The volatile oil profile in different prickly ash pericarps. ZA, samples with green pericarps (*n* = 17) derived from species of *Z. armatum*; ZB1, samples with red pericarps represented by Hancheng (*n* = 29) derived from species of *Z. bungeanum*; ZB2, samples with red pericarps represented by Fengxian (*n* = 13) derived from species of *Z. bungeanum*; Others, samples with red pericarps, a clade of several species (*n* = 13), not derived from species of *Z. bungeanum*.

The hydrodistillation of dry prickly ash pericarps produced odorous clear oils. A total of 47 volatile constituents in the oils were detected in most of the prickly ash pericarps. Among these volatile constituents, 28 constituents were monoterpenes, including 10 chain monoterpenes, 11 monocyclic monoterpenes, 5 dicyclic monoterpenes, and 2 tricyclic monoterpenes; 15 constituents were sesquiterpenes, including 2 chain sesquiterpenes, 5 monocyclic sesquiterpenes, 6 dicyclic sesquiterpenes, 1 tricyclic sesquiterpene, and 1 tetracyclo sesquiterpene; 15 constituents belonged to other types or derivates of monoterpenes and sesquiterpenes (Table S6). The volatile oil profile in the prickly ash pericarps from different plantations was diverse, but the volatile oil profile in pericarps from the same prickly ash species was similar (Figure 1).

#### 3.1.2. Difference of Volatile Oil Profile in Different Prickly Ash Pericarps

To differentiate the volatile oil profile among different prickly ash pericarps, 16 dominating constituents in the volatile oils of the prickly ash pericarps were quantitatively detected. As shown in Table 1, the difference in the contents of sabinene in pericarp volatile oils among the four groups was not significant, while the differences in the contents of other constituents were significant (*p* < 0.05). The most abundant constituents were D-limonene (ZA, 55.1 mg/mL; ZB1, 172.9 mg/mL; ZB2, 158.3 mg/mL; others, 175.4 mg/mL), alfa-myrcene (ZA, 25.3 mg/mL; ZB1, 147.2 mg/mL; ZB2, 115.7 mg/mL; others, 118.1 mg/mL), and linalool (ZA, 256.5 mg/mL; ZB1, 35.5 mg/mL; ZB2, 77.7 mg/mL; others, 52.3 mg/mL). The contents of alfa-myrcene, alfa-ocimene, linalyl acetate, geranyl acetate, D-limonene, gamma-terpinene, terpinolene, terpinen-4-ol, terpineol, alfa-terpinyl acetate, and alfa-pinene in ZA pericarp volatile oils were the lowest among the four prickly ash groups, while the contents of linalyl acetate and geranyl acetate in ZB2 pericarp volatile oils were the highest among the four prickly ash groups. Moreover, the content of linalool in ZB2 pericarp volatile oils was significantly higher than that in ZB1 pericarp volatile oils, but the contents of alfa-terpinyl acetate, alfa-pinene, beta-elemene, and caryophyllene in ZB1 pericarp volatile oils were significantly higher than those in ZB2 pericarp volatile oils.

### 3.2. Chemometric Analyses for Prickly Ash Pericarps Based on Volatile Oil Profile

The volatile oil profile of prickly ash pericarps varied in content and constituents among different groups and different plantations. To better understand the volatile oil profile of different prickly ash pericarps and determine the important differential volatile oil constituents between pericarps from different groups, several chemometric analysis methods were conducted based on the volatile oil profile in prickly ash pericarps.

#### 3.2.1. Principal Component Analysis (PCA)

**Figure 2:**
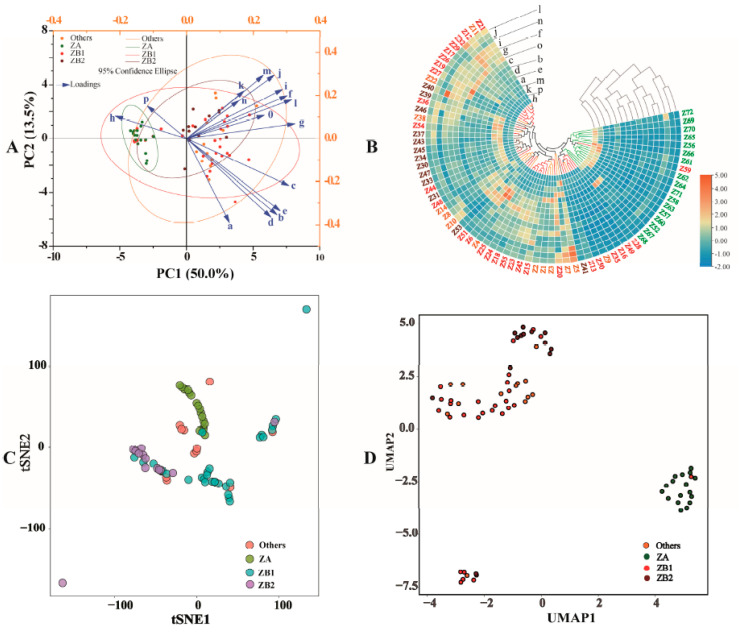
Geographical differentiation of prickly ash pericarps from 72 plantations based on the volatile oil profile of pericarps: (**A**) principal component analysis, (**B**) cluster heat map, (**C**) t-distributed stochastic neighbor embedding, (**D**) uniform manifold approximation and projection for dimension reduction. Sample codes or spots with the same colors represent the samples that belong to the same group; PC, principal component; ZA, samples with green pericarps (*n* = 17) derived from species of *Z. armatum*; ZB1, samples with red pericarps represented by Hancheng (*n* = 29) derived from species of *Z. bungeanum*; ZB2, samples with red pericarps represented by Fengxian (*n* = 13) derived from species of *Z. bungeanum*; others, samples with red pericarps, a clade of several species (*n* = 13), not derived from species of *Z. bungeanum*; tSNE, t-distributed stochastic neighbor embedding; UMAP, uniform manifold approximation and projection for dimension reduction; a, sabinene; b, alfa-myrcene; c, D-limonene; d, alfa-pinene; e, alfa-ocimene; f, gamma-terpinene; g, terpinolene; h, linalool; i, terpinen-4-ol; j, terpineol; k, linalyl acetate; l, alfa-terpinyl acetate; m, geranyl acetate; n, caryophyllene; o, beta-elemene; p, nerolidol.

First, as an unsupervised pattern recognition method, PCA was well able to reveal the sample distribution of prickly ash pericarps from 72 plantations (Figure 2A). Four PCs whose eigenvalue roots were greater than one were generated from the original data, accounting for 83.8% of the variation (50.0%, 13.5%, 11.3%, and 9.0%) and 16.2% of the loss information. Among 16 volatile oil constituents contributed to two principal components, geranyl acetate, terpinol, terpinen-4-ol, gamma-terpinene, alfa-terpinyl acetate, terpinolene, D-limonene, alfa-myrcene, alfa-ocimene, alfa-pinene, and sabinene were far from the origin of coordinates and contributed more to the variation of each axis. The constituents gamma-terpinene, alfa-terpinyl acetate, terpinolene, and D-limonene contributed more to the variation of PC1, while alfa-myrcene, alfa-ocimene, alfa-pinene, and sabinene contributed more to the variation of PC2. Thus, these highly contributing constituents could be used to distinguish pericarps from different groups, and they may be the key constituents in evaluating the quality of prickly ash pericarps. Moreover, the distribution state of different pericarp samples was not in tune with the previous groups based on the results of ITS2 and GBS simplified genome sequencing. ZA samples were clustered together, but some samples from different plantations in ZB and others groups were conjoint.

#### 3.2.2. Cluster Heat Map (CHM)

Another unsupervised pattern recognition method, CHM, was used to classify the 72 pericarp samples and to observe the differences in the volatile oil profile in the pericarps from different plantations (Figure 2B). Nerolidol and linalool clustered into the first group, linalyl acetate and geranyl acetate clustered into the second group, and the rest of the volatile oil constituents clustered into the third group. The volatile oil constituents in the same group shared a similar synthesis and accumulation pathway of the constituents in the pericarps. All samples were classified into three groups: the first group contained most of the ZA pericarps, Z56 (Hanyuan, Sichuan), Z57 (Wusheng, Sichuan), Z58 (Dazhou, Sichuan), Z59 (Pengxi, Sichuan), Z60 (Lezhi, Sichuan), Z61 (Jinyang, Sichuan), Z62 (Jiangjin, Chongqing), Z63 (Qianjiang, Chongqing), Z64 (Fengdu, Chongqing), Z65 (Fengdu, Chongqing), Z66 (Yongshan, Yunnan), Z69 (Qixingguan, Guizhou), Z70 (Guanling, Guizhou), Z71 (Zhenfeng, Guizhou), and Z72 (Xiushui, Jiangxi); the second group contained 11 samples, Z9 (Pingshan, Hebei), Z13 (Pingshun, Shanxi), Z16 (Lingbao, Henan), Z28 (Chunhua, Shaanxi), Z35 (Xunhua, Qinghai), Z41 (Qinan, Gansu), Z49 (Mianning, Sichuan), Z50 (Yanyuan, Sichuan), Z52 (Qiaojiaxian, Yunnan), Z67 (Ludian, Yunnan), and Z68 (Qiaojiaxian, Yunnan); the third group contained the rest of the samples. Similarly to the result of prickly ash pericarp sample distribution in PCA method, the clusters of samples from different plantations were not consistent with the results based on ITS2 and GBS simplified genome sequencing. Some samples from different species were clustered into the same group.

#### 3.2.3. t-Distributed Stochastic Neighbor Embedding (tSNE)

tSNE, a nonlinear model, was used to project the volatile oil data of prickly ash pericarps to a two-dimensional coordinate system, which could reduce the volatile oil data and visualize the distribution of pericarp samples (Figure 2C). The spots in the figure represent the distribution of prickly ash pericarp samples, reflecting the similarity between the samples. The closer the spots, the greater the similarity of the volatile oil profile in these pericarps. Some pericarp samples from different groups were distributed more closely, while most of the samples from the same species were found together.

#### 3.2.4. Uniform Manifold Approximation and Projection for Dimension Reduction (UMAP)

Another nonlinear model, UMAP tended to better retain the global structure of data and visualized the distribution of pericarp samples (Figure 2D). Four discriminant groups (ZA, ZB1, ZB2, and others) were generated based on the genetic background. The spots in the figure represent the distribution of prickly ash pericarp samples, reflecting the similarity between the samples. The closer the spots, the greater the similarity of the volatile oil profile in these pericarps. Some samples from different species were distributed more closely, while most of the samples from the same species were found together.

#### 3.2.5. Orthogonal Partial Least Squares Discriminant Analysis (OPLS-DA)

Since volatile oil constituents in the pericarps varied in different groups (ZA, ZB1, ZB2, and Others), OPLS-DA was applied to determine the important differential indicators to distinguish each group (Table 2). The explanatory rates of the model for ZA vs. ZB1, ZA vs. ZB2, ZA vs. others were more than 0.5 based on R^2^X, R^2^Y, and Q^2^Y: the predictive abilities of this model for these groupings were better. The verification results confirmed that the discrimination of ZA was more accurate compared to the other groups. The influence of intensity and the explanatory ability of each indicator on the discrimination of each group can be measured via the values of variables important in projection (VIP). The indicators included in the model were important when the value of VIP was no less than one. The VIP values varied in distinguishing pericarps from different groups. The VIP values of volatile oil constituents, nine constituents between ZA pericarps and ZB1 pericarps (alfa-myrcene, alfa-ocimene, linalool, D-limonene, gamma-terpinene, terpinolene, terpinen-4-ol, alfa-terpinyl acetate, and nerolidol), 10 constituents between ZA pericarps and ZB2 pericarps (alfa-ocimene, linalool, linalyl acetate, geranyl acetate, D-limonene, gamma-terpinene, terpinolene, terpinen-4-ol, terpineol, and alfa-terpinyl acetate), eight constituents between ZA pericarps and other pericarps (alfa-myrcene, alfa-ocimene, linalool, D-limonene, terpinolene, terpineol, alfa-terpinyl acetate, and alfa-pinene), were more than one. Four constituents between ZB1 pericarps and ZB2 pericarps (linalyl acetate, geranyl acetate, terpineol, and beta-elemene), four constituents between ZB1 pericarps and other pericarps (geranyl acetate, terpineol, nerolidol, and caryophyllene), and six constituents between ZB2 pericarps and other pericarps (linalyl acetate, geranyl acetate, alfa-pinene, nerolidol, beta-elemene, and caryophyllene) were more than one, but the predictive abilities of this model for these groupings were not good, based on R^2^Y (ZB1 vs. others, 0.34) and Q^2^Y (ZB1 vs. ZB2, 0.34; ZB1 vs. others, −0.001; ZB2 vs. others, 0.27).

#### 3.2.6. Discriminant Analysis (DA)

**Figure 3:**
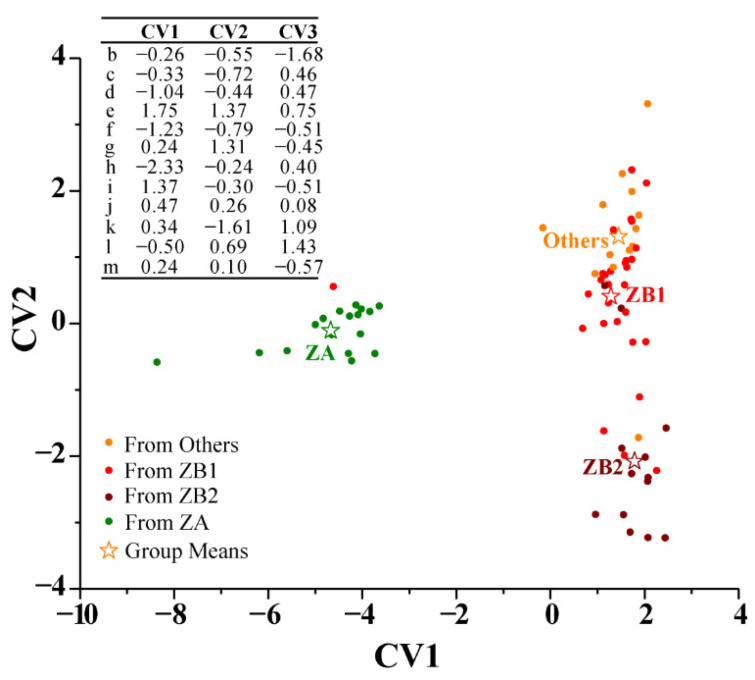
Discriminant analysis of prickly ash pericarps from 72 plantations based on volatile oil profiling of pericarps. Sample codes or spots with the same colors represent the samples belonging to the same group; CV represents canonical variable; ZA, samples with green pericarps (*n* = 17) derived from species of *Z. armatum*; ZB1, samples with red pericarps represented by Hancheng (*n* = 29) derived from species of *Z. bungeanum*; ZB2, samples with red pericarps represented by Fengxian (*n* = 13) derived from species of *Z. bungeanum*; others, samples with red pericarps, a clade of several species (*n* = 13), not derived from species of *Z. bungeanum*; b, alfa-myrcene; c, D-limonene; d, alfa-pinene; e, alfa-ocimene; f, gamma-terpinene; g, terpinolene; h, linalool; i, terpinen-4-ol; j, terpineol; k, linalyl acetate; l, alfa-terpinyl acetate; m, geranyl acetate.

DA, a supervised analysis, confirmed a discrimination model to classify the pericarp samples (Figure 3). The number of variables could be not more than the number of samples in each group in the discrimination model. Due to the lower contribution of sabinene in distinguishing pericarps from different groups and the low contents of nerolidol, beta-elemene, and caryophyllene in pericarp volatile oils, 12 volatile oil constituents were chosen as variables before running the program (canonical discriminant analysis system). The prior probability was selected equally, and the discrimination function was selected linearly. For the discrimination function, alfa-ocimene, linalool, gamma-terpinene, and terpinen-4-ol in the pericarp volatile oils contributed the most to the first canonical variable (CV1), alfa-ocimene, linalyl acetate, and terpinolene in the pericarp volatile oils contributed the most to the second canonical variable (CV2), alfa-myrcene, linalyl acetate, and alfa-terpinyl acetate contributed the most to the third canonical variable (CV3). The discrimination was as follows: the values of 12 volatile oil constituents were substituted into three equations, and the unknown samples were compared to the means of the canonical variable obtained from the model, CV1 (−4.67 for ZA, 1.29 for ZB1, 1.79 for ZB2, and 1.45 for others), CV2 (−0.11 for ZA, 0.41 for ZB1, −2.08 for ZB2, and 1.31 for others), and CV3 (0.05 for ZA, −0.75 for ZB1, 0.42 for ZB2, and1.19 for others); subsequently, the unknown samples were classified into a certain group. An error rate of 25.93% (0.00% for ZA, 34.48% for ZB1, 23.08% for ZB2, and 45.15% for others) was generated from the discrimination functions using cross-validation. The discrimination of ZA was more accurate compared to the other groups, which is similar to the result of OPLS-DA.

### 3.3. The Influences of Environmental Factors on Volatile Oil Profile

The differences in the volatile oil profile of prickly ash pericarps were identified for different groups. Consequently, the influence of environmental factors that affect volatile oil profiling needs to be explored. The filtering of environmental variables was done using the procedure of interactive forward selection (Table S6). Of these environmental variables, soil Pb (pseudo-F = 9.1, *p* = 0.002), soil N_t_ (pseudo-F = 2.7, *p* = 0.03), soil P_t_ (pseudo-F = 4.1, *p* = 0.01), and soil As (pseudo-F = 4.2, *p* = 0.01) significantly influenced volatile oil variations in ZA pericarps, and soil Pb was the key influence factor with higher explanatory and contributing abilities (37.8% for Pb, 6.0% for N_t_, 6.8% for P_t_, and 3.8% for As). The Long of the plantation (pseudo-F = 3.2, *p* = 0.04) and soil Mn (pseudo-F = 3.0, *p* = 0.05) were the key influence factors that caused volatile oil variations in ZB1 pericarps with higher explanatory and contributing abilities (10.6% for Long and 9.3% for Mn). Soil K_a_ (pseudo-F = 4.1, *p*= 0.00), AtP of the plantation (pseudo-F = 3.5, *p* = 0.03), soil OM (pseudo-F = 4.3, *p* = 0.03), and soil Pb (pseudo-F = 2.4, *p* = 0.04) significantly influenced volatile oil variations in ZB2 pericarps, and soil Ka was the key influence factor with higher explanatory and contributing abilities (27.2% for soil K_a_, 6.0% for soil N_t_, 18.7% for AtP, 17.5% for OM, and 8.3 % for soil Pb).

**Figure 4:**
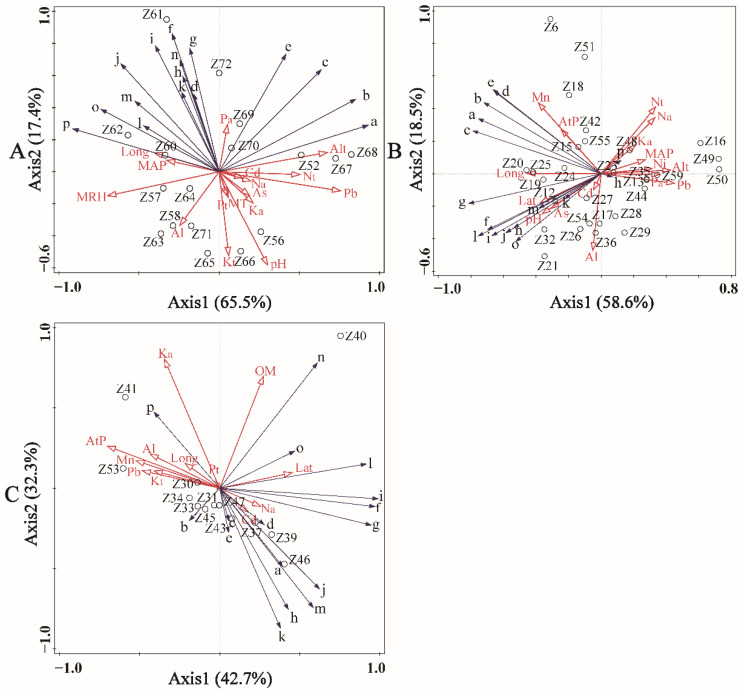
The relationships between environmental factors and volatile oil profiling in prickly ash pericarps: (**A**) ZA, (**B**) ZB1, (**C**) ZB2. ZA, samples with green pericarps (n = 17) derived from species of Z, armatum; ZB1, samples with red pericarps represented by Hancheng (n = 29) derived from species of Z. bungeanum; ZB2, samples with red pericarps represented by Fengxian (n = 13) derived from species of Z. bungeanum; Lat represents latitude of the plantation; Long represents longitude of the plantation; Alt represents altitude of the plantation; AtP represents mean atmospheric pressure of the plantation; MAP represents mean annual precipitation of the plantation; MRH represents mean relative humidity of the plantation; MT represents mean temperature of the plantation; pH represents power of hydrogen in the soil; OM represents organic matter content in the soil; Nt represents the total nitrogen content in the soil; Pt represents the total phosphorus content in the soil; Kt represents the total potassium content in the soil; Na represents available nitrogen content in the soil; Pa represents the available phosphorus content in the soil; Ka represents the available phosphorus content in the soil; Al represents aluminum content in the soil; As represents arsenic content in the soil; Cd represents cadmium content in the soil; Mn represents manganese content in the soil; Ni represents nickel content in the soil; Pb represents lead content in the soil; a, sabinene; b, alfa-myrcene; c, D-limonene; d, alfa-pinene; e, alfa-ocimene; f, gamma-terpinene; g, terpinolene; h, linalool; i, terpinen-4-ol; j, terpineol; k, linalyl acetate; l, alfa-terpinyl acetate; m, geranyl acetate; n, caryophyllene; o, beta-elemene; p, nerolidol.

As shown in Figure 4, 82.9% of the total variation in the volatile oil profile of the ZA pericarps (the explained portions of the first two RDA axes were 65.5% and 17.4%, respectively), 77.1% of the total variation in the volatile oil profile of the ZB1 pericarps (the explained portions of the first two RDA axes were 58.6% and 18.5%, respectively), and 76.0% of the total variation in the volatile oil profile of the ZB1 pericarps (the explained portions of the first two RDA axes were 41.7% and 34.3%, respectively), were explained by selective environmental variables for each group. The degree of influence of environmental factors is expressed by the arrow lengths in the figure: longer arrows represent greater influence on variations of the volatile oil profile in pericarps. Moreover, the relationships between two variables are represented by the cosine values of two arrows, and relationships between the samples are represented by the distances between two sites [27]. The distributions of the samples in each group were dispersive, indicating that selective environmental variables significantly influenced the volatile oil profile of the prickly ash pericarps in the model. For the key environmental influence factors in each group, soil Pb and N_t_ reduced nerolidol and beta-elemene in the ZA pericarp volatile oils significantly, soil P_t_ reduced contents of terpineol, beta-elemene, and alfa-terpinyl acetate in the ZA pericarp volatile oils significantly (Figure 4A); the Long of the plantation increased the contents of terpineol and D-limonene in the ZB1 pericarp volatile oils significantly, and reduced the content of nerolidol in the ZB1 pericarp volatile oils significantly. Soil Mn increased the contents of alfa-ocimene, alfa-pinene, alfa-myrcene, sabinene, and D-limonene in the ZB1 pericarp volatile oils significantly, and reduced the content of linalool in the ZB1 pericarp volatile oils significantly (Figure 4B); soil K_a_ increased the content of nerolidol in the ZB2 pericarp volatile oils significantly, and reduced the contents of alfa-pinene, sabinene, terpinen-4-ol, geranyl acetate, linalool, and linalyl acetate in the ZB2 pericarp volatile oils significantly (Figure 4C).

## 4. Discussion

As a primary processing product, the volatile oils of prickly ash pericarps perform increasingly important roles in the food, pharmacological, and industrial fields [13]. The different content and compositions of volatile oils in prickly ash pericarps contribute to diverse flavors when used as spices and vary in bioactivity when used as functional food or pharmacological material. The content difference of volatile oils underlies different qualities and prices of pericarps. The difference in the content of volatile oils in prickly ash pericarps among different plantations and different groups (ZA, 6.02 mL/100 g; ZB1, 2.90 mL/100 g; ZB2, 4.60 mL/100 g; others, 2.26 mL/100 g) was reported in our previous article [9]. In this continuous research, a total of 47 constituents were detected in the volatile oils of prickly ash pericarps. To ensure the accuracy of this constituent identification, the constituents were identified by comparing their mass spectra fragmentation with NIST and by comparing RI with the results reported in the literature. Moreover, the identified constituents were found in most samples and the components had high similarities (similarity index and reverse similarity index) and probability. Some main constituents were identified by comparing their mass spectra fragmentation with authentic standards. The volatile oil profile in the prickly ash pericarps from different plantations was diverse. The most abundant constituents in volatile oils of prickly ash pericarps were D-limonene, alfa-myrcene, and linalool. The volatile oils of ZA pericarps were linalool odor type, in which D-limonene in volatile oils was significantly higher than that in other groups and most of the other components were significantly lower than those in other groups. D-limonene has been generally found in pericarps from other *Zanthoxylum* species, and this constituent contributed the most to the volatile oils [18]. The volatile oils of ZB pericarps were of the limonene odor type, in which D-limonene in volatile oils was significantly higher than that in ZA pericarps. The volatile oils of ZB2 pericarps were of the acetate odor type, in which linalyl acetate and geranyl acetate in volatile oils were significantly higher than that in other groups. For the two members in ZB species, the content of linalool in ZB2 pericarp volatile oils was significantly higher than that in ZB1 pericarp volatile oils, but the contents of alfa-terpinyl acetate, alfa-pinene, beta-elemene, and caryophyllene in ZB1 pericarp volatile oils were significantly higher than those in ZB2 pericarp volatile oils. In general, genotype and environmental variations affected volatile oil synthesis in the plants [4,20]. Moreover, the differences in volatile oils in prickly ash pericarps were caused by the sample harvest time and extraction method [2,28]. The prickly ash pericarps in this study were collected at the period of commodity maturity when some pericarps in the trees had cracked, and the sampled pericarps were dried and extracted using the same treatment conditions. Thus, the volatile oil profiling variations in prickly ash pericarps were influenced by prickly ash species and environmental conditions of the plantations.

ZA, ZB, and some other *Zanthoxylum* species are botanically related and they are often confused due to their similarities in morphological characteristics, especially in ZB species. Moreover, the overlapping distribution of some prickly ash species and inconsistent nomenclature of the species cultivated in different areas make discrimination of similar pericarps more difficult. The volatile oil profile coupled with chemometrics has been widely used to identify potential indicators to distinguish and classify samples obtained from different species and from different origins [16,20]. Therefore, chemometric analyses based on volatile oil in prickly ash pericarps were conducted. Six chemometric methods (PCA, CHM, tSNE, UMAP, OPLS-DA, and DA) were carried out to better understand the differences in volatile oil profiling in pericarps from different plantations, and RDA was used to determine the key environmental factors that cause volatile oil variations. PCA and CHM were unsupervised models to reveal linear relations between each indicator [29,30], while tSNE and UMAP were nonlinear models for reducing indicators and clustering the samples [31,32]. ZA samples were obviously clustered together, but some of the samples from different plantations in the ZB and others groups were conjoint. The distributions of samples in these four models’ classification results did not agree with the previously determined groups based on the results of ITS2 and GBS simplified genome sequencing [9,10], indicating that volatile oil profiling in pericarps, apart from plant species, was also affected by factors in the environment in which the species are grown. The variables with high negative or positive component loadings contributed more to the variation in each axis in PCA model [33]. The constituents geranyl acetate, terpinol, terpinen-4-ol, gamma-terpinene, alfa-terpinyl acetate, terpinolene, D-limonene, alfa-myrcene, alfa-ocimene, alfa-pinene, and sabinene were representative components for distinguishing the pericarp samples. The constituents in the same group shared a similar synthesis and accumulation pathway to the component in the CHM model [34]. Nerolidol and linalool share a similar synthesis and accumulation pathway, linalyl acetate and geranyl acetate share another similar synthesis and accumulation pathway, and the rest of the volatile oil constituents share other synthesis and accumulation pathways in the pericarps. The tSNE and UMAP models well demonstrated the state of ZA pericarps. The constituents were important differential indicators to distinguish each group in the OPLS-DA model when the explanatory rates (R^2^X, R^2^Y, and Q^2^Y) were more than 0.4 and the VIP value was no less than one [35]. The verification results confirmed that the discrimination of ZA was more accurate compared to the other groups: nine constituents (alfa-myrcene, alfa-ocimene, linalool, D-limonene, gamma-terpinene, terpinolene, terpinen-4-ol, alfa-terpinyl acetate, and nerolidol) were important differential indicators to distinguish pericarps from ZA and ZB1; 10 constituents (alfa-ocimene, linalool, linalyl acetate, geranyl acetate, D-limonene, gamma-terpinene, terpinolene, terpinen-4-ol, terpineol, and alfa-terpinyl acetate) were important differential indicators to distinguish pericarps from ZA and ZB2; eight constituents (alfa-myrcene, alfa-ocimene, linalool, D-limonene, terpinolene, terpineol, alfa-terpinyl acetate, and alfa-pinene) were important differential indicators to distinguish pericarps from ZA and others. The rest of the constituents were not important differential indicators to distinguish pericarps, with lower explanatory rates in distinguishing groups or a lower VIP of the indicators. DA provided a good approach to distinguishing ZA pericarps based on volatile oil profiling.

ZB1 and ZB2 with red pericarps are widely planted species in north China, and ZA with green pericarps is mainly distributed in south China. ZB1 and ZB2 are often confused because of the similarity in morphological characteristics and even have the same genotype [10]. The differences in volatile oil profiling among different groups were investigated. More detailed relationships between environmental factors and volatile oil variations were highlighted in the RDA model. The key influence factors causing volatile oil variations were diverse for different groups (soil Pb, N_t_, P_t_, and As for ZA pericarp volatile oils; Long of the plantation and soil Mn for ZB1 pericarp volatile oils; Soil K_a_, OM, Pb, and AtP of the plantation for ZB2 pericarp volatile oils). Some toxic elements (soil Pb and As) affected the volatile oil profiling in pericarps. To some extent, soil Pb reduced the contents of nerolidol and beta-elemene in ZA pericarp volatile oils, and reduced the contents of gamma-terpinene, terpinolene, and terpineol in ZB2 pericarp volatile oils. Fortunately, a previous study has revealed that the contents of the toxic elements As and Pb were extremely low in pericarp volatile oils [36], and hence using pericarp volatile oils poses no health risk.

## 5. Conclusions

In this study, 47 constituents were detected in the volatile oils of prickly ash pericarps. The most abundant constituents were D-limonene, alfa-myrcene, and linalool in volatile oils of prickly ash pericarps. The volatile oil profile of prickly ash pericarps was affected by the species of the plant and some environmental factors, and the key environmental factors that affected volatile profiling variations for different prickly ash species were diverse. Chemometrics based on the volatile oil profile could properly distinguish ZA pericarps from other pericarps. The results of this study provide comprehensive information on the volatile oil profile of pericarps from different prickly ash species and from different plantations, and they determined important volatile oil constituents to distinguish prickly ash species. Moreover, this study analyzed the relationships between the volatile oil profile and environmental factors for ZA and ZB pericarps, and speculated on the key environmental factors that cause volatile oil variations for each species. It can also be beneficial in evaluating systems of pericarp quality and helping to obtain better prickly ash pericarp volatile oils by introducing better cultivars and improving the cultivated environments.

## Figures and Tables

**Table 1:** The volatile oil profile in different prickly ash pericarps.

Component	ZA	ZB1	ZB2	Others
Alfa-myrcene	25.3 ± 17.8 b	147.2 ± 98.4 a	115.7 ± 66.5 a	118.1 ± 56.4 a
Alfa-ocimene	3.5 ± 1.7 b	38.4 ± 27.4 a	30.9 ± 14.9 a	44.1 ± 24.3 a
Linalool	256.5 ± 54.2 a	35.5 ± 26.1 c	77.7 ± 26.3 b	52.3 ± 30.4 c
Linalyl acetate	0.8 ± 0.5 c	5.4 ± 4.9 b	15.2 ± 5.6 a	6.5 ± 5.2 b
Geranyl acetate	1.1 ± 1.0 c	11.5 ± 9.9 b	23.1 ± 10.8 a	18.0 ± 13.2 ab
D-limonene	55.1 ± 31.0 b	172.9 ± 97.0 a	158.3 ± 49.3 a	175.4 ± 82.0 a
Gamma-terpinene	10.1 ± 4.9 b	52.5 ± 30.9 a	47.6 ± 22.8 a	42.2 ± 25.8 a
Terpinolene	2.8 ± 1.3 b	10.5 ± 6.0 a	9.5 ± 4.1 a	10.4 ± 4.9 a
Terpinen-4-ol	17.1 ± 7.9 b	78.2 ± 47.0 a	72.1 ± 34.3 a	63.2 ± 38.5 a
Terpineol	12.3 ± 5.7 b	46.4 ± 30.8 a	60.6 ± 23.0 a	72.5 ± 55.8 a
Alfa-terpinyl acetate	1.7 ± 2.3 c	35.8 ± 23.0 ab	28.7 ± 12.0 b	47.8 ± 25.1 a
Sabinene	40.5 ± 37.5	40.0 ± 28.2	33.3 ± 17.6	56.0 ± 41.1
Alfa-pinene	1.2 ± 0.9 c	28.1 ± 22.6 a	15.2 ± 7.2 b	35.3 ± 25.8 a
Nerolidol	5.1 ± 3.8 a	0.3 ± 0.4 c	0.3 ± 0.4 c	2.7 ± 3.7 b
Beta-elemene	1.1 ± 0.9 b	6.2 ± 5.0 a	1.4 ± 1.0 b	6.0 ± 3.7 a
Caryophyllene	4.7 ± 2.3 ab	4.9 ± 3.3 ab	3.6 ± 6.0 b	7.8 ± 4.6 a

Result is reported as means ± standard deviations of triplicate measurements; The different letters (a, b, c, or ab) behind the result indicate significant differences among different prickly ash groups, no letters behind the result indicate no significant difference among different prickly ash groups (*p* < 0.05); The letter “ab” behind the result indicates significant difference compared with “a” or “b” behind the result (*p* < 0.05), but it indicates no significant difference compared with “a” or “b” behind the result (*p* < 0.01); ZA, samples with green pericarps (*n* = 17) derived from species of *Z. armatum*; ZB1, samples with red pericarps represented by Hancheng (*n* = 29) derived from species of *Z. bungeanum*; ZB2, samples with red pericarps represented by Fengxian (*n* = 13) derived from species of *Z. bungeanum*; others, samples with red pericarps, a clade of several species (*n* = 13), not derived from *Z. bungeanum*.

**Table 2:** Variables important in projection and verification by permutation test between different prickly ash groups.

Indicators	ZA vs. ZB1	ZA vs. ZB2	ZA vs. Others	ZB1 vs. ZB2	ZB1 vs. Others	ZB2 vs. Others
Sabinene	0.02	0.21	0.49	0.26	0.43	0.80
Alfa-myrcene	1.05	0.94	1.01	0.55	0.23	0.23
D-limonene	1.02	1.01	1.08	0.16	0.39	0.39
Alfa-pinene	0.97	0.80	1.17	0.86	0.40	1.39
Alfa-ocimene	1.03	1.02	1.21	0.43	0.45	0.85
Gamma-terpinene	1.13	1.04	0.95	0.17	0.84	0.46
Terpinolene	1.09	1.02	1.11	0.01	0.14	0.03
Linalool	1.63	1.31	1.37	0.94	0.85	0.87
Terpinen-4-ol	1.09	1.02	0.94	0.14	0.93	0.42
Terpineol	0.85	1.11	1.03	1.16	1.74	0.11
Linalyl acetate	0.69	1.52	0.79	2.39	0.62	2.05
Alfa-terpinyl acetate	1.17	1.01	1.28	0.16	0.90	0.89
Geranyl acetate	0.83	1.35	0.98	1.97	1.57	1.07
Caryophyllene	0.04	0.26	0.59	0.83	1.42	1.44
Beta-elemene	0.95	0.12	0.95	1.45	0.43	1.38
Nerolidol	1.18	0.99	0.55	0.21	2.01	1.09
R^2^X	0.70	0.70	0.69	0.64	0.57	0.48
R^2^Y	0.83	0.94	0.90	0.51	0.34	0.67
Q^2^Y	0.78	0.91	0.85	0.34	−0.001	0.27

R^2^X represents the explanatory rate for the X matrix in the model; R^2^Y represents the explanatory rate for the Y matrix in the model; Q^2^Y represents the predictive ability of the model. In theory, the model is better when R^2^X, R^2^Y, and Q^2^Y are closer to one; usually, the model is better if R^2^X, R^2^Y, and Q^2^Y are higher than 0.5, and it is acceptable if R^2^X, R^2^Y, and Q^2^Y are higher than 0.4; ZA, samples with green pericarps (*n* = 17) derived from species of *Z. armatum*; ZB1, samples with red pericarps represented by Hancheng (*n* = 29) derived from species of *Z. bungeanum*; ZB2, samples with red pericarps represented by Fengxian (*n* = 13) derived from species of *Z. bungeanum*; others, samples with red pericarps, a clade of several species (*n* = 13), not derived from species of *Z. bungeanum*.

## Data Availability

Not applicable.

## Supplementary Materials

The following are available online at www.mdpi.com/2304-8158/10/10/2386/s1, Table S1 Information on group, location, and climate for the 72 prickly ash pericarp samples. ZA, samples with green pericarps (*n* = 17) derived from species of *Z. armatum*; ZB1, samples with red pericarps represented by Hancheng (*n* = 29) derived from species of *Z. bungeanum*; ZB2, samples with red pericarps represented by Fengxian (*n* = 13) derived from species of *Z. bungeanum*; others, samples with red pericarps, a clade of several species (*n* = 13), not derived from *Z. bungeanum*. Table S2 Qualitative and quantitative determinations of each volatile oil constituent. y, peak area; x, the content of each constituent. Table S3 Z-Score values of location and climate for the 72 prickly ash pericarp samples. ZA, samples with green pericarps (*n* = 17) derived from species of *Z. armatum*; ZB1, samples with red pericarps represented by Hancheng (*n* = 29) derived from species of *Z. bungeanum*; ZB2, samples with red pericarps represented by Fengxian (*n* = 13) derived from species of *Z. bungeanum*; others, samples with red pericarps, a clade of several species (*n* = 13), not derived from *Z. bungeanum*. Table S4 Z-Score values of soil conditions in different prickly ash plantations. ZA, samples with green pericarps (*n* = 17) derived from species of *Z. armatum*; ZB1, samples with red pericarps represented by Hancheng (*n* = 29) derived from species of *Z. bungeanum*; ZB2, samples with red pericarps represented by Fengxian (*n* = 13) derived from species of *Z. bungeanum*; others, samples with red pericarps, a clade of several species (*n* = 13), not derived from *Z. bungeanum*. Table S5 Z-Score values of the volatile oil profile in different prickly ash pericarps. a, sabinene; b, alfa-myrcene; c, D-limonene; d, alfa-pinene; e, alfa-ocimene; f, gamma-terpinene; g, terpinolene; h, linalool; i, terpinen-4-ol; j, terpineol; k, linalyl acetate; l, alfa-terpinyl acetate; m, geranyl acetate; n, caryophyllene; o, beta-elemene; p, nerolidol; ZA, samples with green pericarps (*n* = 17) derived from species of *Z. armatum*; ZB1, samples with red pericarps represented by Hancheng (*n* = 29) derived from species of *Z. bungeanum*; ZB2, samples with red pericarps represented by Fengxian (*n* = 13) derived from species of *Z. bungeanum*; others, samples with red pericarps, a clade of several species (*n* = 13), not derived from *Z. bungeanum*. Table S6 Identification of constituents in prickly ash pericarp volatile oils. Table S7 The influences of environmental factors on the volatile oil profile in prickly ash pericarps. ZA, samples with green pericarps (*n* = 17) derived from species of *Z. armatum*; ZB1, samples with red pericarps represented by Hancheng (*n* = 29) derived from species of *Z. bungeanum*; ZB2, samples with red pericarps represented by Fengxian (*n* = 13) derived from species of *Z. bungeanum*; Lat represents latitude of the plantation; Long represents longitude of the plantation; Alt represents altitude of the plantation; AtP represents mean atmospheric pressure of the plantation; MAP represents mean annual precipitation of the plantation; MRH represents mean relative humidity of the plantation; MT represents mean temperature of the plantation; pH represents power of hydrogen in the soil; OM represents organic matter content in the soil; N_t_ represents the total nitrogen content in the soil; P_t_ represents the total phosphorus content in the soil; K_t_ represents the total potassium content in the soil; N_a_ represents available nitrogen content in the soil; P_a_ represents the available phosphorus content in the soil; K_a_ represents the available phosphorus content in the soil; Al represents aluminum content in the soil; As represents arsenic content in the soil; Cd represents cadmium content in the soil; Mn represents manganese content in the soil; Ni represents nickel content in the soil; Pb represents lead content in the soil.

## Abbreviations

ZA (*n* = 17) represents green pericarps derived from *Zanthoxylum armatum*; ZB1 (*n* = 29) represents red pericarps derived from *Z. bungeanum* from Hancheng; ZB2 (*n* = 13) represents red pericarps derived from *Z. bungeanum* from Fengxian; Others (*n* = 13) includes the rest of the samples that have red pericarps excluding *Z. bungeanum*; PC, principal component; tSNE, t-distributed stochastic neighbor embedding; UMAP, uniform manifold approximation and projection for dimension reduction; R^2^X represents the explanatory rate for the X matrix in the model; R^2^Y represents the explanatory rate for the Y matrix in the model; Q^2^Y represents the predictive ability of the model; CV represents canonical variable; Lat represents latitude of the plantation; Long represents longitude of the plantation; Alt represents altitude of the plantation; AtP represents mean atmospheric pressure of the plantation; MAP represents mean annual precipitation of the plantation; MRH represents mean relative humidity of the plantation; MT represents mean temperature of the plantation; pH represents power of hydrogen in the soil; OM represents organic matter content in the soil; N_t_ represents the total nitrogen content in the soil; P_t_ represents the total phosphorus content in the soil; K_t_ represents the total potassium content in the soil; N_a_ represents available nitrogen content in the soil; P_a_ represents the available phosphorus content in the soil; K_a_ represents the available phosphorus content in the soil; Al represents aluminum content in the soil; As represents arsenic content in the soil; Cd represents cadmium content in the soil; Mn represents manganese content in the soil; Ni represents nickel content in the soil; Pb represents lead content in the soil.
