# Supplementary material for: Volatile Oil Profile of Prickly Ash (Zanthoxylum) Pericarps from Different Locations in China
Source: Foods. 2021 Oct 8;10(10):2386. doi: 10.3390/foods10102386 (PMC8535335; doi:10.3390/foods10102386)
Supplement: Supplementary file 1 [file foods-10-02386-s001.zip › foods-1385935-supplementary.pdf]

Supplementary tables

Volatile Oil Profile of Prickly Ash Pericarps from Different Locations in China

Yao Ma [1,2], Lu Tian [1,2], Xiaona Wang [1,2], Chen Huang [1], Mingjing Tian [1], and Anzhi Wei [1,2,*]

[1] College of Forestry, Northwest A&F University, Yangling 712100, China; mayao277000@nwafu.edu.cn (Y.M.); t1anlu@nwafu.edu.cn (L.T.); 17709590958@nwafu.edu.cn (X.W.); hc19990513@nwafu.edu.cn (C.H.); 15531971892@nwafu.edu.cn (M.T.); weianzhi@nwafu.edu.cn (A.W.)

[2] Research Centre for Engineering and Technology of *Zanthoxylum*, State Forestry Administration, Yangling 712100, China

* Correspondence: weianzhi@nwafu.edu.cn; Tel.: +86-029-8708-2211

Supplementary tables

Table S1 Information of group, location, and climate for the 72 prickly ash pericarp samples.
ZA, samples with green pericarps (n=17) derived from species of *Z, armatum*; ZB1, samples with red pericarps represented by Hancheng (n=29) derived from species of *Z. bungeanum*; ZB2, samples with red pericarps represented by Fengxian (n=13) derived from species of *Z. bungeanum*; Others, samples with red pericarps, a clade of several species (n=13), not derive from *Z. bungeanum*.
Table S2 Qualitative and quantitative determinations of each volatile oil constituent.
y, peak area; x, the content of each constituent.
Table S3 Z-Score values of location and climate for the 72 prickly ash pericarp samples.
ZA, samples with green pericarps (n=17) derived from species of *Z, armatum*; ZB1, samples with red pericarps represented by Hancheng (n=29) derived from species of *Z. bungeanum*; ZB2, samples with red pericarps represented by Fengxian (n=13) derived from species of *Z. bungeanum*; Others, samples with red pericarps, a clade of several species (n=13), not derive from *Z. bungeanum*.
Table S4 Z-Score values of soil conditions in different prickly ash plantations.
ZA, samples with green pericarps (n=17) derived from species of *Z, armatum*; ZB1, samples with red pericarps represented by Hancheng (n=29) derived from species of *Z. bungeanum*; ZB2, samples with red pericarps represented by Fengxian (n=13) derived from species of *Z. bungeanum*; Others, samples with red pericarps, a clade of several species (n=13), not derive from *Z. bungeanum*.
Table S5 Z-Score values of volatile oil profile in different prickly ash pericarps.
a, Sabinene; b, Alfa-myrcene; c, D-limonene; d, Alfa-pinene; e, Alfa-ocimene; f, Gamma-terpinene; g, Terpinolene; h, Linalool; i, Terpinen-4-ol; j, Terpineol; k, Linalyl acetate; l, Alfa-terpinyl acetate; m, Geranyl acetate; n, Caryophyllene; o, Beta-elemene; p, Nerolido; ZA, samples with green pericarps (n=17) derived from species of *Z, armatum*; ZB1, samples with red pericarps represented by Hancheng (n=29) derived from species of *Z. bungeanum*; ZB2, samples with red pericarps represented by Fengxian (n=13) derived from species of *Z. bungeanum*; Others, samples with red pericarps, a clade of several species (n=13), not derive from *Z. bungeanum*.
Table S6 Identification of constituents in prickly ash pericarp volatile oils.
Table S7 The influences of environmental factors on volatile oil profile in prickly ash pericarps.
ZA, samples with green pericarps (n=17) derived from species of *Z, armatum*; ZB1, samples with red pericarps represented by Hancheng (n=29) derived from species of *Z. bungeanum*; ZB2, samples with red pericarps represented by Fengxian (n=13) derived from species of *Z. bungeanum*; Lat represents latitude of the plantation; Long represents longitude of the plantation; Alt represents altitude of the plantation; AtP represents mean atmospheric pressure of the plantation; MAP represents mean annual precipitation of the plantation; MRH represents mean relative humidity of the plantation; MT represents mean temperature of the plantation; pH represents power of hydrogen in the soil; OM represents organic matter content in the soil; N_t represents the total nitrogen content in the soil; P_t represents the total phosphorus content in the soil; K_t represents the total potassium content in the soil; N_a represents available nitrogen content in the soil; P_a represents the available phosphorus content in the soil; K_a represents the available phosphorus content in the soil; Al represents aluminium content in the soil; As represents arsenic content in the soil; Cd represents cadmium content in the soil; Mn represents manganese content in the soil; Ni represents nickel content in the soil; Pb represents lead content in the soil.

foods

Table S1 Information of group, location, and climate for the 72 prickly ash pericarp samples

Group	Code	Origin Province	Origin County	Longitude(°)	Latitude(°)	Altitude (m)	Atmospheric pressure (hPa)	Mean temperature (°C)	Mean relative humidity (%)	Mean annual precipitation (mm)
Others	Z1	Shandong	Yiyuan	118.33	36.03	210	981.7	12.5	62	689.5
Others	Z2	Shandong	Laicheng	117.66	36.20	192	990.2	13.3	62	695.3
Others	Z3	Shandong	Shanting	117.59	35.06	195	1008.0	14.4	66	820.2
Others	Z4	Hebei	Laishui	115.45	39.67	298	1013.2	12.2	62	531
Others	Z5	Hebei	Tangxian	114.78	38.86	191	1008.9	12.7	65	508.1
Others	Z7	Hebei	Shexian	113.66	36.55	433	961.8	12.8	61	535
Others	Z8	Hebei	Fuping	114.20	38.85	274	983.4	12.6	56	572.1
Others	Z9	Hebei	Pingshan	114.20	38.25	138	983.4	12.6	56	572.1
Others	Z10	Shanxi	Wutai	113.26	38.73	1032	892.4	7.0	59	503.7
Others	Z11	Shanxi	Xiaoyi	111.78	37.15	753	928.0	11.0	57	448.5
Others	Z14	Shanxi	Yangcheng	112.41	35.49	628	940.8	12.0	61	578.4
Others	Z22	Shaanxi	Huanglong	109.72	35.60	1138	893.8	9.0	63	566.2
Others	Z38	Gansu	Dongxiang	103.24	35.74	1807	759.5	5.6	62	534.9
ZB1	Z6	Hebei	Shexian	113.66	36.55	433	961.8	12.8	61	535
ZB1	Z12	Shanxi	Zaoning	110.66	35.80	835	907.1	10.5	59	515
ZB1	Z13	Shanxi	Pingshun	113.65	36.36	578	893.6	9.4	59	553
ZB1	Z15	Shanxi	Ruicheng	110.69	34.69	504	957.9	13.0	67	505.3
ZB1	Z16	Henan	Lingbao	110.59	34.50	692	961.7	13.5	66	600.5
ZB1	Z17	Henan	Hubin	111.19	34.77	358	969.1	14.4	61	549.6
ZB1	Z18	Henan	Linzhou	113.94	36.26	396	980.8	13.2	66	648.9
ZB1	Z19	Henan	Baofeng	112.90	33.93	212	1000.7	14.6	69	747.2
ZB1	Z20	Shaanxi	Yanchang	110.41	36.51	968	924.6	10.4	62	480.8

ZB1	Z21	Shaanxi	Yichuan	110.37	35.94	793	921.0	10.3	61	516
ZB1	Z23	Shaanxi	Hancheng	110.47	35.55	452	935.0	12.1	64	535.4
ZB1	Z24	Shaanxi	Heyang	110.32	35.30	549	935.0	12.1	64	535.4
ZB1	Z25	Shaanxi	Huazhou	109.72	34.43	741	975.3	13.7	71	577.5
ZB1	Z26	Shaanxi	Fuping	109.12	34.82	526	961.6	12.1	68	636.5
ZB1	Z27	Shaanxi	Yaozhou	109.03	34.94	820	934.5	12.7	62	543.4
ZB1	Z28	Shaanxi	Chunhua	108.66	34.75	737	902.0	10.6	67	585.7
ZB1	Z29	Shaanxi	Chunhua	108.66	34.75	737	902.0	10.6	67	585.7
ZB1	Z32	Shaanxi	Chencang	106.45	34.53	915	946.1	13.5	66	645.8
ZB1	Z35	Qinghai	Xunhua	102.49	35.85	1870	812.6	9.0	54	271.8
ZB1	Z36	Gansu	Jishishan	103.08	35.68	1841	808.1	7.3	67	501.2
ZB1	Z42	Gansu	Gangu	105.34	34.73	1300	874.0	10.7	68	437.3
ZB1	Z44	Gansu	Xihe	105.29	33.74	1086	842.4	9.1	73	527.9
ZB1	Z48	Sichuan	Hanyuan	102.64	29.35	939	923.9	17.8	69	755.6
ZB1	Z49	Sichuan	Mianning	102.19	28.29	1654	820.6	14.1	70	1106.7
ZB1	Z50	Sichuan	Yiyuan	101.51	27.42	2563	999.0	12.3	60	828.6
ZB1	Z51	Yunnan	Yongping	99.54	25.46	1667	999.0	15.8	75	951.7
ZB1	Z54	Guizhou	Zhenfeng	105.63	25.61	1224	894.5	16.6	79	1318.3
ZB1	Z55	Guizhou	Qixingguan	105.31	27.30	1511	848.7	13.0	81	866.1
ZB1	Z59	Sichuan	Pengxi	105.71	30.76	366	969.4	16.8	81	929.1
ZB2	Z30	Shaanxi	Yangling	108.01	34.30	528	904.8	12.1	68	636.5
ZB2	Z31	Shaanxi	Chencang	106.70	34.53	1050	946.1	13.5	66	645.8
ZB2	Z33	Shaanxi	Taibai	107.12	33.99	1401	846.5	8.1	70	712.7
ZB2	Z34	Shaanxi	Fengxian	106.65	33.97	1061	904.8	12.1	68	636.5
ZB2	Z37	Gansu	Yongjing	103.29	35.96	1633	835.3	9.7	59	273.8
ZB2	Z39	Gansu	Linxia	103.15	35.75	1789	808.1	7.3	67	501.2

ZB2	Z40	Gansu	Jingning	105.73	35.52	1668	834.4	7.8	66	414
ZB2	Z41	Gansu	Qin'an	105.80	35.06	1404	880.1	10.9	66	436.3
ZB2	Z43	Gansu	Lixian	105.05	33.79	1473	860.3	10.3	71	470.7
ZB2	Z45	Gansu	Wudu	110.47	35.55	452	893.6	14.9	58	460.8
ZB2	Z46	Sichuan	Songpan	103.60	32.66	2865	720.9	6.3	63	708.3
ZB2	Z47	Sichuan	Maoxian	103.85	31.68	1602	840.6	11.2	74	462.3
ZB2	Z53	Yunnan	Ludian	103.23	27.31	1356	999.0	12.2	76	852.8
ZA	Z52	Yunnan	Qiaojiaxian	103.15	27.35	1733	916.2	20.8	60	838.2
ZA	Z56	Sichuan	Hanyuan	102.39	29.68	1641	923.9	17.8	69	755.6
ZA	Z57	Sichuan	Wusheng	106.30	30.35	288	977.5	17.5	84	1051.1
ZA	Z58	Sichuan	Daxian	107.25	31.19	478	974.4	17.2	80	1205.1
ZA	Z60	Sichuan	Lezhi	104.95	30.21	424	972.7	17.3	81	859.8
ZA	Z61	Sichuan	Jinyang	103.25	27.70	1288	791.6	15.9	70	795
ZA	Z62	Chongqing	Jiangjin	106.12	29.06	250	984.0	18.3	81	1000.9
ZA	Z63	Chongqing	Qianjiang	106.65	29.03	235	945.6	15.7	79	1172.8
ZA	Z64	Chongqing	Fengdu	107.71	30.22	397	980.3	18.3	80	1018.7
ZA	Z65	Chongqing	Fengdu	107.71	30.22	397	980.3	18.3	80	1018.7
ZA	Z66	Yunan	Yongshan	103.63	28.23	844	804.8	16.6	74	684.8
ZA	Z67	Yunan	Ludian	103.23	27.31	1356	999.0	12.2	76	852.8
ZA	Z68	Yunan	Qiaojiaxian	102.98	27.36	1324	916.2	20.8	60	838.2
ZA	Z69	Guizhou	Qixingguan	105.31	27.30	1511	848.7	13.0	81	866.1
ZA	Z70	Guizhou	Guanling	105.66	25.68	591	887.0	16.4	79	1327.6
ZA	Z71	Guizhou	Zhenfeng	105.63	25.61	1224	894.5	16.6	79	1318.3
ZA	Z72	Jiangxi	Xiushui	114.16	29.06	151	998.9	16.8	79	1601.8

ZA, samples with green pericarps (n=17) derived from group of *Z, armatum*; ZB1, samples with red pericarps represented by Hancheng (n=29) derived from group of *Z. bungeanum*; ZB2, samples with red pericarps represented by Fengxian (n=13) derived from group of *Z. bungeanum*; Others, samples with red pericarps, a clade of several species (n=13), not derive from *Z. bungeanum.*

foods

MDPI

Table S2 Qualitative and quantitative determinations of each volatile oil component

Retention time	Component	Cas	Regression equation	Regression coefficient (R^2)	Range (mg/mL)
15.13	Sabinene	3387-41-5	$y = 4*10^9x - 50255$	0.9999	0-211.0
16.17	Alfa-myrcene	123-35-3	$y = 2*10^9x - 5*10^6$	0.9989	0-558.2
17.98	D-limonene	5989-27-5	$y = 6*10^9x + 2*10^6$	0.9999	0-568.1
18.53	Alfa-pinene	80-56-8	$y = 7*10^9x + 5*10^6$	0.9997	0-171.6
19.03	Alfa-ocimene	13877-91-3	$y = 3*10^9x + 5*10^6$	0.9997	0-402.0
19.50	Gamma-terpinene	99-85-4	$y = 3*10^9x - 3*10^6$	0.9997	0-170.0
20.96	Terpinolene	586-62-9	$y = 5*10^9x + 5*10^6$	0.9999	0-215.0
21.59	Linalool	78-70-6	$y = 1*10^{10}x - 6*10^6$	0.9996	0-500.0
25.27	Terpinen-4-ol	562-74-3	$y = 5*10^9x + 2*10^6$	1.0000	0-232.3
25.9	Terpineol	98-55-5	$y = 3*10^9x + 3*10^7$	0.9808	0-250.0
28.93	Linalyl acetate	115-95-7	$y = 5*10^{10}x - 6*10^6$	0.9996	0-50.0
33.14	Alfa-terpinyl acetate	80-26-2	$y = 4*10^9x - 6*10^6$	0.9998	0-237.5
34.63	Geranyl acetate	105-87-3	$y = 4*10^9x - 4*10^6$	0.9997	0-230.0
36.15	Caryophyllene	87-44-5	$y = 1*10^{10}x - 8*10^6$	0.9992	0-45.1
35.00	Beta-elemene	515-13-9	$y = 9*10^9x - 7*10^6$	0.9989	0-50.0
41.89	Nerolidol	40716-66-3	$y = 7*10^9x - 1*10^7$	0.9963	0-50.0

y, peak area; x, the content of each component.

Table S3 Z-Score values of location and climate for the 72 prickly ash pericarp samples

Group	Code	Longitude	Latitude	Altitude	Atmospheric pressure	Mean temperature	Mean relative humidity	Mean annual precipitation
Others	Z1	2.34	0.79	-1.19	0.93	-0.15	-0.77	-0.06
Others	Z2	2.19	0.84	-1.22	1.06	0.09	-0.77	-0.04
Others	Z3	2.17	0.54	-1.21	1.32	0.42	-0.25	0.44
Others	Z4	1.69	1.74	-1.04	1.40	-0.24	-0.77	-0.66
Others	Z5	1.54	1.53	-1.22	1.34	-0.09	-0.38	-0.75
Others	Z7	1.28	0.93	-0.82	0.64	-0.06	-0.90	-0.65
Others	Z8	1.41	1.53	-1.08	0.96	-0.12	-1.55	-0.51
Others	Z9	1.41	1.37	-1.31	0.96	-0.12	-1.55	-0.51
Others	Z10	1.19	1.50	0.17	-0.40	-1.77	-1.16	-0.77
Others	Z11	0.86	1.08	-0.29	0.13	-0.59	-1.42	-0.98
Others	Z14	1.00	0.65	-0.50	0.32	-0.29	-0.90	-0.48
Others	Z22	0.39	0.68	0.34	-0.38	-1.18	-0.64	-0.53
Others	Z38	-1.07	0.72	1.45	-2.38	-2.19	-0.77	-0.65
ZB1	Z6	1.28	0.93	-0.82	0.64	-0.06	-0.90	-0.65
ZB1	Z12	0.61	0.73	-0.16	-0.18	-0.74	-1.16	-0.72
ZB1	Z13	1.28	0.88	-0.58	-0.38	-1.06	-1.16	-0.58
ZB1	Z15	0.61	0.44	-0.70	0.58	0.00	-0.12	-0.76
ZB1	Z16	0.59	0.39	-0.39	0.63	0.15	-0.25	-0.40
ZB1	Z17	0.73	0.46	-0.94	0.74	0.42	-0.90	-0.59
ZB1	Z18	1.35	0.85	-0.88	0.92	0.06	-0.25	-0.21
ZB1	Z19	1.11	0.24	-1.19	1.21	0.48	0.13	0.16
ZB1	Z20	0.55	0.92	0.06	0.08	-0.77	-0.77	-0.85
ZB1	Z21	0.54	0.77	-0.23	0.03	-0.80	-0.90	-0.72

ZB1	Z23	0.56	0.67	-0.79	0.24	-0.26	-0.51	-0.65
ZB1	Z24	0.53	0.6	-0.63	0.24	-0.26	-0.51	-0.65
ZB1	Z25	0.39	0.37	-0.31	0.84	0.21	0.39	-0.49
ZB1	Z26	0.26	0.47	-0.67	0.63	-0.26	0.01	-0.26
ZB1	Z27	0.24	0.51	-0.18	0.23	-0.09	-0.77	-0.62
ZB1	Z28	0.15	0.46	-0.32	-0.26	-0.71	-0.12	-0.46
ZB1	Z29	0.15	0.46	-0.32	-0.26	-0.71	-0.12	-0.46
ZB1	Z32	-0.34	0.4	-0.02	0.40	0.15	-0.25	-0.23
ZB1	Z35	-1.24	0.74	1.55	-1.59	-1.18	-1.80	-1.65
ZB1	Z36	-1.11	0.7	1.50	-1.65	-1.68	-0.12	-0.78
ZB1	Z42	-0.60	0.45	0.61	-0.67	-0.68	0.01	-1.02
ZB1	Z44	-0.61	0.19	0.26	-1.14	-1.15	0.65	-0.68
ZB1	Z48	-1.21	-0.96	0.01	0.07	1.42	0.13	0.19
ZB1	Z49	-1.31	-1.23	1.20	-1.47	0.33	0.26	1.53
ZB1	Z50	-1.46	-1.46	2.70	1.19	-0.21	-1.03	0.47
ZB1	Z51	-1.91	-1.97	1.22	1.19	0.83	0.91	0.94
ZB1	Z54	-0.53	-1.94	0.49	-0.37	1.07	1.43	2.34
ZB1	Z55	-0.60	-1.49	0.96	-1.05	0.00	1.69	0.61
ZB1	Z59	-0.51	-0.73	-0.93	0.75	1.13	1.69	0.85
ZB2	Z30	0.01	0.34	-0.66	-0.21	-0.26	0.01	-0.26
ZB2	Z31	-0.29	0.4	0.20	0.40	0.15	-0.25	-0.23
ZB2	Z33	-0.19	0.26	0.78	-1.08	-1.45	0.26	0.03
ZB2	Z34	-0.30	0.25	0.22	-0.21	-0.26	0.01	-0.26
ZB2	Z37	-1.06	0.77	1.16	-1.25	-0.97	-1.16	-1.64
ZB2	Z39	-1.09	0.72	1.42	-1.65	-1.68	-0.12	-0.78
ZB2	Z40	-0.51	0.66	1.22	-1.26	-1.54	-0.25	-1.11

ZB2	Z41	-0.49	0.54	0.78	-0.58	-0.62	-0.25	-1.02
ZB2	Z43	-0.66	0.21	0.90	-0.88	-0.80	0.39	-0.89
ZB2	Z45	0.56	0.67	-0.79	-0.38	0.56	-1.29	-0.93
ZB2	Z46	-0.99	-0.09	3.19	-2.95	-1.98	-0.64	0.01
ZB2	Z47	-0.93	-0.35	1.11	-1.17	-0.53	0.78	-0.93
ZB2	Z53	-1.07	-1.49	0.70	1.19	-0.24	1.04	0.56
ZA	Z52	-1.09	-1.48	1.33	-0.04	2.31	-1.03	0.51
ZA	Z56	-1.26	-0.87	1.17	0.07	1.42	0.13	0.19
ZA	Z57	-0.38	-0.69	-1.06	0.87	1.33	2.07	1.32
ZA	Z58	-0.16	-0.48	-0.75	0.82	1.24	1.56	1.91
ZA	Z60	-0.68	-1.39	-0.84	0.80	1.27	1.69	0.59
ZA	Z61	-1.07	-1.03	0.59	-1.90	0.86	0.26	0.34
ZA	Z62	-0.42	-1.04	-1.12	0.97	1.57	1.69	1.13
ZA	Z63	-0.30	-0.73	-1.15	0.39	0.80	1.43	1.78
ZA	Z64	-0.06	-0.73	-0.88	0.91	1.57	1.56	1.20
ZA	Z65	-0.06	-1.25	-0.88	0.91	1.57	1.56	1.20
ZA	Z66	-0.98	-1.49	-0.14	-1.70	1.07	0.78	-0.08
ZA	Z67	-1.07	-1.48	0.70	1.19	-0.24	1.04	0.56
ZA	Z68	-1.13	-1.49	0.65	-0.04	2.31	-1.03	0.51
ZA	Z69	-0.60	-1.92	0.96	-1.05	0.00	1.69	0.61
ZA	Z70	-0.52	-1.94	-0.56	-0.48	1.01	1.43	2.37
ZA	Z71	-0.53	-1.03	0.49	-0.37	1.07	1.43	2.34
ZA	Z72	1.40	-1.48	-1.29	1.19	1.13	1.43	3.42

ZA, samples with green pericarps (n=17) derived from group of *Z, armatum*; ZB1, samples with red pericarps represented by Hancheng (n=29) derived from group of *Z. bungeanum*; ZB2, samples with red pericarps represented by Fengxian (n=13) derived from group of *Z. bungeanum*; Others, samples with red pericarps, a clade of several species (n=13), not derive from *Z. bungeanum.*

foods MDPI

Table S4 Z-Score values of soil conditions in different prickly ash plantations

Group	Code	pH	OM	N_t	P_t	K_t	N_a	P_a	K_a	Al	As	Cd	Pb	Mn	Ni
Others	Z1	0.02	0.52	0.72	-0.72	0.49	0.06	-0.67	-0.30	-0.17	1.06	-0.03	0.01	0.25	-0.2
Others	Z2	-0.34	-0.20	-0.18	0.68	0.60	-0.33	2.06	-1.01	1.18	-1.16	-0.78	0.44	0.06	-0.21
Others	Z3	-0.70	0.40	0.35	-0.51	-0.22	0.07	-0.29	-0.60	0.39	0.44	0.01	0.94	1.42	1.73
Others	Z4	0.29	1.12	0.89	-0.10	-0.07	0.07	0.13	0.87	0.67	-0.8	-0.42	0.14	-0.43	-0.71
Others	Z5	0.38	0.70	0.44	0.13	-0.41	0.13	0.15	1.96	-1.1	-0.14	-0.46	-0.24	-0.01	-0.42
Others	Z7	0.54	-0.25	-0.44	-0.78	-0.60	-0.30	-0.70	-0.49	-1.24	0.5	-0.35	-0.38	-0.27	-0.06
Others	Z8	0.80	-0.68	-0.74	0.12	-0.81	-0.26	0.15	1.63	-1.31	0.53	-0.2	-0.38	-0.2	-0.21
Others	Z9	0.21	-0.29	-0.13	-0.52	0.02	0.00	-0.21	-0.26	-1.59	0.39	-0.27	-0.34	0.09	0.03
Others	Z10	-0.01	0.67	0.93	-0.11	0.04	0.16	-0.48	-0.16	-1.05	0.75	-0.02	-0.35	0.09	-0.39
Others	Z11	0.68	-0.52	-0.73	-0.67	-0.78	-0.56	-0.70	-0.56	-0.48	0.17	-0.38	-0.46	-0.41	-0.48
Others	Z14	0.02	3.48	3.08	-0.43	-0.64	1.33	-0.24	1.19	-0.89	0.86	2.61	2.16	-0.42	-0.27
Others	Z22	0.43	-0.03	-0.01	-0.60	-1.19	-0.19	-0.55	-0.34	-1.49	0.3	-0.35	-0.54	-0.45	-0.45
Others	Z38	0.79	-0.77	-0.84	-0.55	0.11	-0.53	-0.64	-0.85	-0.61	0.56	-0.19	-0.49	-0.35	-0.41
ZB1	Z6	0.50	0.13	0.15	-0.67	-0.08	-0.21	-0.71	-0.44	-1.03	-0.2	-0.6	-0.46	0.34	-0.13
ZB1	Z12	0.35	-0.63	-0.65	-0.48	-0.99	-0.44	-0.28	-0.67	0.04	0.34	-0.42	-0.49	-0.4	-0.43
ZB1	Z13	0.86	0.11	0.07	0.75	1.43	-0.09	-0.65	-0.70	0.12	0.43	-0.31	-0.42	-0.85	0.02
ZB1	Z15	0.63	-0.84	-0.88	-0.49	-0.42	-0.83	-0.70	1.11	-0.92	0.07	-0.41	-0.53	-0.64	-0.62
ZB1	Z16	0.54	-0.74	-0.74	-0.36	-1.16	-0.46	-0.60	-0.05	-0.74	-0.04	0.04	0.5	-0.54	-0.55
ZB1	Z17	0.74	-0.73	-0.81	-0.78	-0.57	-0.60	-0.69	-0.28	0.27	0.07	-0.31	-0.43	-0.54	-0.44
ZB1	Z18	0.33	-0.53	-0.91	-0.93	-0.36	-0.63	-0.60	-0.17	-1.27	0.55	-0.16	-0.36	0.57	0.66
ZB1	Z19	0.69	-0.95	-1.09	-0.52	-0.65	-0.76	-0.66	-0.86	-1.12	0.39	-0.3	-0.44	0.74	0.2
ZB1	Z20	0.67	-0.93	-0.90	-0.66	-0.83	-0.54	-0.61	-0.53	-0.06	0.06	-0.48	-0.61	-0.59	-0.67
ZB1	Z21	0.97	-0.92	-1.05	-0.87	-0.72	-0.70	-0.71	-0.83	1.51	0.18	-0.4	-0.58	-0.46	-0.34

ZB1	Z23	0.51	0.48	-0.27	-0.41	-0.95	-0.42	-0.45	-0.48	-0.93	0.18	-0.29	-0.2	-0.5	-0.42
ZB1	Z24	0.80	-0.59	-0.66	-0.12	-0.84	-0.47	0.18	0.81	-0.14	0.33	-0.22	-0.41	-0.24	-0.33
ZB1	Z25	0.66	-0.06	-0.47	-0.45	-0.89	-0.45	-0.14	0.17	-0.54	0.25	-0.26	-0.3	-0.37	-0.37
ZB1	Z26	0.10	-0.55	-0.41	-0.54	-0.91	0.00	0.82	0.84	0.3	0.57	-0.13	-0.31	-0.16	-0.11
ZB1	Z27	0.78	-0.55	-0.57	-0.48	-0.82	-0.40	-0.62	-1.18	-0.31	0.14	-0.3	-0.45	-0.35	-0.29
ZB1	Z28	0.71	-0.61	-0.65	-0.50	-0.93	-0.44	-0.59	-0.98	-0.44	0.21	-0.27	-0.49	-0.34	-0.35
ZB1	Z29	0.57	-0.61	-0.61	-0.45	-0.71	-0.37	-0.53	-0.89	0.04	0.37	-0.19	-0.46	-0.28	-0.26
ZB1	Z32	0.45	-0.71	-0.67	-0.40	0.08	-0.53	-0.53	-0.27	0.08	0.4	-0.1	-0.42	-0.16	-0.19
ZB1	Z35	0.53	-0.53	-0.50	-0.40	-0.64	-0.49	-0.56	-0.74	0.4	0.37	-0.38	-0.37	-0.47	-0.19
ZB1	Z36	0.92	-0.79	-0.75	-0.38	-0.55	-0.65	-0.47	-0.34	0.17	0.64	-0.38	-0.45	-0.52	0.12
ZB1	Z42	0.54	-0.57	-0.30	1.10	0.42	-0.30	0.33	1.40	0.54	0.13	-0.27	-0.43	-0.32	-0.32
ZB1	Z44	0.79	-0.97	-0.99	-0.42	0.36	-0.56	0.01	-0.95	0.34	0.68	1.55	0.08	-0.34	-0.37
ZB1	Z48	-2.86	1.23	1.31	2.51	-1.66	0.67	4.14	1.34	1.6	-1.39	-1.09	-0.8	0.52	4.16
ZB1	Z49	-1.63	1.25	1.41	2.88	1.13	0.88	3.98	0.77	-0.77	-2.19	-0.89	2.18	-0.99	-1.01
ZB1	Z50	-1.91	0.85	0.76	0.84	-1.47	0.18	0.49	0.72	-0.43	0.5	-0.63	-0.72	-0.39	5.26
ZB1	Z51	-2.34	3.39	2.86	-0.34	-0.21	1.68	-0.39	-0.27	-1.73	0.86	1.07	-0.65	0.52	-0.3
ZB1	Z54	-2.13	1.11	1.16	2.30	-0.66	0.58	2.87	0.94	1.17	1.53	2.93	1.01	0.09	2.04
ZB1	Z55	0.57	-0.58	-0.49	0.95	0.44	-0.36	0.23	-0.24	1.97	-1.98	-0.88	-0.88	0.16	0.1
ZA	Z59	0.33	-0.36	-0.21	0.18	0.72	-0.28	-0.21	1.15	-0.42	-1.16	-0.35	-0.32	-0.41	-0.21
ZB2	Z30	0.53	-0.50	-0.31	-0.26	-0.70	-0.31	-0.54	0.90	-0.72	0.32	-0.11	-0.37	-0.02	-0.11
ZB2	Z31	0.39	-0.44	-0.32	0.12	-0.13	-0.33	0.30	-0.47	0.5	0.67	-0.01	-0.42	-0.06	-0.02
ZB2	Z33	0.38	-0.41	-0.28	-0.49	-0.66	-0.70	-0.50	-0.84	-0.43	0.26	-0.5	-0.55	-0.53	-0.58
ZB2	Z34	0.67	-0.64	-0.72	-0.27	-0.63	-0.28	-0.62	-0.65	-0.4	0.14	-1.31	0.11	-0.1	-0.22
ZB2	Z37	1.05	-1.02	-1.17	-0.57	-0.08	-0.79	-0.66	-0.79	-1.57	0.38	-0.38	-0.6	-0.47	-0.58
ZB2	Z39	0.62	-0.97	-1.06	-0.43	-0.25	6.72	-0.58	-0.59	-1.32	0.31	-0.3	-0.52	-0.47	-0.55
ZB2	Z40	0.52	1.41	1.80	-0.14	-0.01	0.51	-0.57	1.04	-0.11	0.26	-0.1	-0.36	-0.26	-0.31

ZB2	Z41	0.72	-0.89	-0.94	-0.20	0.28	-0.63	0.00	1.89	0.49	0.43	-0.32	-0.54	-0.38	-0.43
ZB2	Z43	0.47	-0.57	-0.59	1.10	0.59	-0.49	0.60	-0.17	0.72	0.83	-0.08	-0.28	-0.29	-0.38
ZB2	Z45	0.47	-0.23	-0.08	-0.20	0.40	-0.10	-0.04	-1.27	0.54	0.62	-0.22	-0.56	-0.19	-0.31
ZB2	Z46	0.26	-1.17	-1.11	-0.60	0.19	-0.77	-0.73	-1.55	-0.01	2.39	1.23	-0.45	-0.52	0.3
ZB2	Z47	-0.51	0.20	0.51	0.18	0.14	0.30	0.85	-0.19	0.54	0.07	-0.54	-0.58	-0.57	-0.33
ZB2	Z53	-0.57	-0.04	-0.11	-0.35	3.04	0.53	0.24	0.83	0.94	0.17	0.5	3.95	1.57	-0.43
ZA	Z52	-0.68	-0.02	0.11	-0.98	0.76	0.04	-0.72	-0.39	-1.13	-0.84	0.04	1.07	-0.82	-0.03
ZA	Z56	-0.61	3.16	3.53	5.29	0.34	2.37	2.30	2.68	0.29	0.49	0.92	2.85	-0.01	-0.72
ZA	Z57	-2.34	-0.50	-0.10	0.00	-0.93	1.48	0.78	3.19	0.78	-2.36	-0.43	-0.44	-0.41	-0.23
ZA	Z58	-1.07	-0.44	-0.44	-0.86	0.48	0.04	-0.69	-1.28	1.71	-1.77	-0.59	-0.41	-0.39	0.36
ZA	Z60	-0.35	-0.63	-0.41	0.14	0.75	-0.33	-0.52	-0.64	-0.67	-0.87	-0.09	-0.36	0.35	0.08
ZA	Z61	-4.10	0.90	0.86	-0.30	0.41	0.37	1.40	-0.23	-1.42	-1.57	-0.54	0.05	-0.79	-0.64
ZA	Z62	0.16	-0.35	-0.21	2.12	0.99	-0.23	0.24	-0.46	0.81	-1.32	0.04	-0.44	0.21	-0.16
ZA	Z63	0.01	-0.87	-1.07	-0.50	1.74	-0.39	-0.42	-1.04	1.25	-2.15	-0.55	-0.55	-0.05	0.12
ZA	Z64	0.08	-0.61	-0.64	0.09	1.36	-0.31	-0.09	-0.75	1.25	-2.15	-0.55	-0.55	-0.05	0.12
ZA	Z65	0.18	1.33	1.57	0.55	2.52	0.21	-0.24	0.66	0.21	0.28	0.6	1.03	0.11	-0.87
ZA	Z66	0.14	-0.21	-0.12	-0.12	2.89	-0.39	-0.42	-0.66	2.01	-0.78	-0.39	-0.02	0.88	0.51
ZA	Z67	0.16	0.56	0.72	0.08	2.70	-0.09	-0.33	0.00	0.94	0.17	0.5	3.95	1.57	-0.43
ZA	Z68	-0.01	1.77	1.73	0.40	-0.08	0.83	0.70	1.62	-1.41	-2.02	0.39	2.05	1.71	-0.77
ZA	Z69	-0.41	1.41	0.99	-0.22	-0.99	-0.19	-0.67	-1.32	1.97	-1.98	-0.88	-0.88	0.16	0.1
ZA	Z70	-0.04	-0.02	0.64	0.11	-0.40	-0.07	0.60	1.09	1.32	1.87	5.25	0.41	6.99	1.94
ZA	Z71	-1.02	0.43	0.56	-0.46	1.79	0.39	-0.67	0.74	1.17	1.53	2.93	1.01	0.09	2.04
ZA	Z72	-1.88	0.37	0.18	-0.03	-0.62	-0.09	0.61	-0.79	1.73	0.89	0.77	0.11	0.29	-0.06

ZA, samples with green pericarps (n=17) derived from group of *Z, armatum*; ZB1, samples with red pericarps represented by Hancheng (n=29) derived from group of *Z. bungeanum*; ZB2, samples with red pericarps represented by Fengxian (n=13) derived from group of *Z. bungeanum*; Others, samples with red pericarps, a clade of several species (n=13), not derive from *Z. bungeanum.*

Table S5 Z-Score values of volatile oil profile in different prickly ash pericarps

Group	Code	a	b	c	d	e	f	g	h	i	j	k	l	m	n	o	p
Others	Z1	1.79	0.00	0.13	0.16	0.17	-0.43	0.85	-0.63	-0.34	-0.22	0.49	0.30	-0.21	0.35	0.04	-0.57
Others	Z2	2.30	0.21	0.40	0.39	0.38	-0.29	1.23	-0.57	-0.18	-0.05	0.74	0.56	-0.05	0.60	0.22	-0.56
Others	Z3	2.89	0.22	0.31	0.74	0.79	0.09	1.42	-0.59	-0.05	-0.03	0.63	0.49	-0.30	0.80	-0.07	-0.57
Others	Z4	0.74	0.53	-0.21	3.78	2.28	-0.34	-0.25	-0.68	0.06	-0.48	-0.65	-0.08	-0.28	0.05	0.85	-0.56
Others	Z5	0.10	1.00	1.65	1.09	1.32	0.78	0.98	-0.27	0.58	4.21	0.10	3.02	2.09	3.75	1.23	3.17
Others	Z7	-0.93	-0.54	-0.07	-0.33	-0.38	0.29	0.03	-0.37	0.46	2.77	0.09	2.16	2.05	1.40	1.60	1.34
Others	Z8	0.46	0.34	1.09	0.96	0.89	-0.34	-0.21	0.02	-0.66	0.85	-0.41	0.60	1.42	1.22	-0.31	0.90
Others	Z9	-1.22	-1.16	-1.49	-0.87	-1.08	-1.27	-1.46	-0.93	-1.29	-1.04	-0.87	-0.97	-0.76	-1.04	-0.89	-0.37
Others	Z10	0.64	1.14	2.04	1.63	1.53	-0.87	-0.68	-0.64	-0.85	1.52	-0.60	1.37	0.18	-0.14	-0.54	0.72
Others	Z11	0.14	0.14	0.78	0.38	0.10	1.52	1.07	-0.74	1.59	0.84	-0.44	1.34	-0.28	0.16	1.36	-0.49
Others	Z14	0.60	0.80	0.87	1.39	1.63	-0.37	-0.07	-0.54	-0.53	-0.13	-0.26	-0.03	0.23	0.82	0.15	1.38
Others	Z22	-0.83	-0.32	-0.14	-0.08	-0.17	1.39	0.95	-0.73	1.33	-0.33	-0.32	0.62	-0.23	0.35	1.92	-0.55
Others	Z38	-0.91	-0.49	-0.35	-0.30	-0.03	1.18	0.87	0.16	1.10	1.44	2.13	0.96	2.40	0.11	0.24	-0.53
ZB1	Z6	1.35	2.23	1.83	4.19	3.51	0.18	0.94	-0.76	-0.19	-0.37	-0.48	-0.08	-0.27	0.15	0.17	0.01
ZB1	Z12	0.22	0.08	0.62	0.05	-0.10	1.72	1.12	-0.75	1.90	1.45	-0.44	1.46	-0.14	0.74	0.66	-0.24
ZB1	Z13	-1.15	-1.04	-1.13	-0.74	-0.92	-0.38	-0.78	-0.89	-0.29	-0.44	-0.66	-0.21	-0.64	-0.20	-0.01	-0.58
ZB1	Z15	0.40	0.81	1.23	0.18	0.00	0.78	0.42	-0.59	1.97	0.90	-0.50	0.99	-0.56	-0.28	0.08	-0.57
ZB1	Z16	-1.28	-1.18	-1.52	-0.88	-1.10	-1.24	-1.45	-1.03	-1.20	-1.15	-0.95	-1.08	-0.99	-1.18	-0.87	-0.59
ZB1	Z17	0.34	0.91	0.71	0.68	0.80	1.13	0.95	-0.82	0.87	0.01	-0.42	0.37	-0.43	0.93	1.61	-0.57
ZB1	Z18	0.42	2.17	0.76	1.18	1.21	0.50	0.28	-0.90	0.61	-0.71	-0.79	0.24	-0.57	-0.60	0.18	-0.59
ZB1	Z19	1.52	0.89	0.56	1.00	1.06	1.26	1.35	-0.75	1.02	-0.26	-0.32	0.54	-0.36	1.06	1.85	-0.54
ZB1	Z20	1.82	2.26	2.27	1.49	1.37	-0.24	2.03	-0.75	-0.40	1.50	0.79	1.92	2.29	-0.68	0.50	-0.44
ZB1	Z21	-0.41	0.17	0.58	0.60	0.46	1.33	1.15	-0.69	1.47	0.82	-0.14	1.63	0.00	0.59	1.80	-0.55
ZB1	Z23	0.50	1.66	1.66	0.95	1.18	1.34	1.17	-0.69	0.77	0.39	-0.22	0.55	0.00	0.19	0.62	-0.57
ZB1	Z24	0.88	2.00	0.99	0.65	0.64	0.67	0.50	-0.80	0.63	-0.11	-0.69	0.13	-0.53	-0.27	0.46	-0.59

ZB1	Z25	0.30	1.48	0.60	0.38	0.25	0.68	0.33	-0.77	0.73	-0.51	-0.72	0.28	-0.75	-0.47	0.59	-0.49
ZB1	Z26	-0.47	0.23	0.45	0.52	0.39	1.21	0.84	-0.72	1.23	0.20	-0.35	0.77	0.07	1.32	2.23	-0.53
ZB1	Z27	-0.92	-0.45	-0.13	-0.13	-0.14	1.51	0.97	-0.63	1.63	0.01	-0.02	1.19	0.47	1.20	4.03	-0.49
ZB1	Z28	-1.12	-1.00	-1.24	-0.77	-0.96	-0.90	-1.18	-0.98	-0.82	-1.01	-0.86	-0.85	-0.91	-1.10	-0.61	-0.59
ZB1	Z29	0.17	0.80	0.53	0.47	0.39	1.25	0.81	-0.72	1.24	-0.07	-0.17	0.60	-0.15	0.20	2.34	-0.59
ZB1	Z32	0.08	0.45	0.82	0.38	0.56	2.53	2.30	-0.43	2.37	0.97	0.51	2.58	0.60	0.96	1.26	-0.46
ZB1	Z35	-1.29	-1.22	-1.57	-0.93	-1.14	-1.27	-1.47	-1.03	-1.27	-1.18	-0.95	-1.10	-0.99	-1.21	-0.90	-0.59
ZB1	Z36	-0.43	0.22	0.66	0.30	0.42	1.20	1.21	-0.24	0.87	1.47	0.97	0.70	1.59	1.22	0.92	-0.50
ZB1	Z42	0.67	1.12	1.08	0.98	1.10	1.41	1.41	-0.72	1.14	-0.28	-0.37	0.28	-0.36	-0.07	0.48	-0.46
ZB1	Z44	-0.64	-0.11	-0.12	-0.18	0.04	0.17	0.04	-0.32	0.22	0.48	1.60	-0.11	1.08	-0.64	-0.52	-0.57
ZB1	Z48	-0.44	0.08	-0.04	-0.21	0.06	-0.58	-0.28	-0.08	-0.69	0.20	1.24	-0.35	0.97	-0.55	-0.57	-0.54
ZB1	Z49	-1.27	-1.17	-1.51	-0.90	-1.09	-1.31	-1.48	-1.00	-1.33	-1.18	-0.89	-1.11	-0.95	-1.20	-0.93	-0.59
ZB1	Z50	-1.23	-1.10	-1.41	-0.87	-1.08	-1.29	-1.42	-0.80	-1.20	-1.03	-0.77	-1.03	-0.83	-1.17	-0.89	-0.58
ZB1	Z51	0.51	2.03	1.73	1.08	2.23	-0.41	-0.19	-0.15	-0.68	-0.41	0.71	-0.56	0.10	-0.02	-0.26	-0.42
ZB1	Z54	-0.28	-0.11	0.21	-0.27	-0.21	0.67	0.46	-0.16	0.93	0.84	1.44	0.66	0.91	-0.51	0.05	-0.56
ZB1	Z55	0.14	1.29	1.35	0.70	0.90	0.81	0.63	-0.70	0.63	0.39	-0.30	0.31	-0.18	-0.08	0.27	-0.56
ZB1	Z59	-0.59	-0.99	-1.11	-0.89	-1.02	-0.99	-0.64	1.69	-0.87	-0.76	-0.87	-1.07	-0.92	0.05	-0.55	3.09
ZB2	Z30	0.12	-0.30	0.05	-0.66	-0.51	-0.02	-0.25	-0.20	-0.02	0.09	1.48	-0.05	0.54	-0.56	-0.86	-0.56
ZB2	Z31	-0.13	-0.14	0.61	0.17	0.48	0.30	0.32	-0.34	0.10	0.17	1.81	-0.06	0.85	-0.74	-0.70	-0.45
ZB2	Z33	-0.21	-0.18	0.17	-0.61	-0.35	0.31	0.07	-0.20	0.20	0.46	1.53	-0.36	1.09	-1.06	-0.87	-0.57
ZB2	Z34	0.04	0.31	0.31	-0.14	0.08	0.06	-0.06	-0.41	-0.04	-0.06	1.68	-0.31	0.38	-0.97	-0.83	-0.53
ZB2	Z37	-0.40	-0.06	0.13	0.01	0.19	0.40	0.36	0.05	0.89	1.41	1.56	0.44	2.16	-0.48	-0.28	-0.56
ZB2	Z39	1.01	1.04	0.85	0.39	0.86	0.99	1.22	-0.08	0.84	0.70	2.12	0.55	1.49	-0.41	-0.17	-0.57
ZB2	Z40	-0.53	-0.23	0.21	-0.20	-0.07	1.56	1.16	-0.49	1.69	0.11	0.27	1.12	0.18	4.41	-0.35	-0.53
ZB2	Z41	-1.14	-0.87	-0.82	-0.67	-0.86	-0.94	-1.10	-0.86	-0.88	-0.37	-0.77	-0.71	-0.61	-0.41	-0.49	-0.17
ZB2	Z43	-0.97	-0.56	-0.72	-0.45	-0.25	0.36	0.17	0.03	0.49	0.95	1.92	0.28	1.83	-0.85	-0.63	-0.57
ZB2	Z45	-0.30	0.12	0.11	-0.36	-0.22	-0.03	0.06	-0.37	0.07	0.28	1.86	-0.36	0.82	-0.95	-0.78	-0.57

ZB2	Z46	0.06	0.16	0.30	-0.27	0.20	1.35	1.19	0.18	1.25	1.52	2.57	0.21	2.32	-0.89	-0.73	-0.56
ZB2	Z47	-0.23	0.02	0.01	-0.36	-0.11	0.53	0.34	-0.25	0.39	0.46	1.48	-0.09	1.04	-0.88	-0.64	-0.57
ZB2	Z53	-0.77	2.21	1.26	0.06	1.32	-1.16	-1.03	-0.22	-1.14	-0.43	0.68	-0.37	-0.33	-0.93	-0.72	-0.58
ZA	Z52	1.44	-0.59	-0.59	-0.90	-0.93	-1.08	-1.21	1.29	-1.10	-1.05	-0.95	-1.13	-0.99	0.41	-0.90	-0.57
ZA	Z56	0.10	-0.99	-1.03	-0.90	-1.04	-1.15	-1.00	1.59	-1.17	-1.01	-0.83	-1.12	-0.96	-0.44	-0.89	-0.51
ZA	Z57	-0.80	-1.12	-1.27	-0.92	-1.08	-0.99	-1.03	1.37	-0.86	-0.87	-0.91	-1.12	-0.96	-0.30	-0.70	2.02
ZA	Z58	-0.92	-1.15	-1.33	-0.92	-1.09	-1.13	-1.13	1.19	-1.01	-0.92	-0.87	-1.10	-0.94	-0.43	-0.68	1.55
ZA	Z60	-0.73	-1.06	-1.04	-0.89	-1.03	-1.01	-0.96	1.50	-1.02	-0.80	-0.86	-1.05	-0.90	0.02	-0.45	2.11
ZA	Z61	-0.11	-0.96	-0.93	-0.87	-0.96	-0.66	-0.68	3.41	-0.51	-0.53	-0.70	-1.07	-0.85	0.65	-0.48	2.26
ZA	Z62	-1.11	-1.07	-1.21	-0.81	-0.97	-0.79	-1.02	1.36	-0.88	-0.55	-0.77	-0.71	-0.62	0.33	-0.06	2.78
ZA	Z63	-1.17	-1.17	-1.42	-0.91	-1.10	-1.17	-1.30	1.22	-1.09	-0.94	-0.90	-1.11	-0.96	-0.45	-0.64	1.76
ZA	Z64	-0.62	-0.94	-1.03	-0.81	-0.96	-0.92	-0.95	1.72	-0.94	-0.87	-0.85	-1.10	-0.94	-0.50	-0.75	1.34
ZA	Z65	-0.86	-1.12	-1.32	-0.89	-1.07	-1.10	-1.17	1.34	-1.02	-0.96	-0.87	-1.10	-0.96	-0.31	-0.80	0.28
ZA	Z66	-0.47	-1.02	-1.16	-0.87	-1.03	-1.15	-1.25	1.53	-1.16	-0.97	-0.80	-1.12	-0.94	-0.41	-0.87	-0.34
ZA	Z67	2.40	-0.54	-0.47	-0.90	-0.96	-1.07	-1.15	1.25	-1.09	-0.94	-0.89	-1.11	-0.98	-0.66	-0.84	-0.58
ZA	Z68	2.85	-0.55	-0.63	-0.89	-0.96	-1.09	-1.18	1.03	-1.11	-1.06	-0.90	-1.13	-0.98	-0.74	-0.91	-0.51
ZA	Z69	0.34	-0.78	-0.67	-0.79	-0.88	-0.71	-0.66	2.34	-0.65	-0.76	-0.73	-1.07	-0.87	-0.14	-0.79	0.43
ZA	Z70	0.38	-0.91	-0.84	-0.87	-0.97	-0.95	-0.91	2.00	-0.96	-0.84	-0.68	-1.09	-0.89	-0.26	-0.71	0.77
ZA	Z71	-0.84	-1.12	-1.27	-0.91	-1.08	-1.08	-1.17	1.39	-1.05	-0.94	-0.89	-1.11	-0.96	-0.45	-0.70	1.10
ZA	Z72	-0.01	-0.75	-0.14	-0.82	-0.90	-0.71	-0.46	1.28	-0.75	-0.82	-0.82	-1.06	-0.88	1.62	-0.62	0.84

a, Sabinene; b, Alfa-myrcene; c, D-limonene; d, Alfa-pinene; e, Alfa-ocimene; f, Gamma-terpinene; g, Terpinolene; h, Linalool; i, Terpinen-4-ol; j, Terpineol; k, Linalyl acetate; l, Alfa-terpinyl acetate; m, Geranyl acetate; n, Caryophyllene; o, Beta-elemene; p, Nerolidol; ZA, samples with green pericarps (n=17) derived from group of *Z, armatum*; ZB1, samples with red pericarps represented by Hancheng (n=29) derived from group of *Z. bungeanum*; ZB2, samples with red pericarps represented by Fengxian (n=13) derived from group of *Z. bungeanum*; Others, samples with red pericarps, a clade of several species (n=13), not derive from *Z. bungeanum.*

Table S6 Identification of compounds in prickly ash pericarp volatile oils

**Group**	**Compound Name**	**Cas#**	**Molecular**	**Retention index**	**Similarity**	**Probability**	**Proportion**

			Formula	Literature	Experiment	SI	RSI	(%)	(%)
**Other**									
	2-ethyl-oxetane	N	C5H10O		-	803	896	39.14	0.41
	Hydroperoxide, 1-ethylbutyl	24254-56-6	C6H14O2		-	843	851	69.76	0.27
	Hydroperoxide, 1-methylpentyl	24254-55-5	C6H14O2		-	845	864	59	0.25
	Crypton	500-02-7	C9H14O		1099	777	912	48.66	0.18
**Monoterpenoids**									
Chain	Alfa-myrcene	123-35-3	C10H16	991	988	898	902	30.83	4.79
Chain	Alfa-ocimene	13877-91-3	C10H16	1024	1052	936	937	32.93	1.99
Chain	Linalool	78-70-6	C10H18O	1082	1110	946	946	84.28	23.12
Chain	(4E,6E)-allocimene	3016-19-1	C10H16	1043	1142	926	944	34.04	0.7
Chain	D-citronellal	2385-77-5	C10H18O		1168	893	910	37.21	0.12
Chain	cis-geraniol	106-25-2	C10H18O		1248	907	912	48.75	0.28
Chain	Linalyl acetate	115-95-7	C12H20O2	1257	1270	914	917	52.05	5.61
Chain	Alfa-terpinyl acetate	80-26-2	C12H20O2	1347	1366	917	926	39.77	2.65
Chain	Neryl acetate	141-12-8	C12H20O2	1361	1381	915	922	45.44	0.4
Chain	Geranyl acetate	105-87-3	C12H20O2	1362	1400	924	927	42.53	0.95
Monocyclic	Terpinene	99-86-5	C10H16		1015	910	920	13.4	1.44
Monocyclic	D-limonene	5989-27-5	C10H16	1029	1029	891	892	18.85	19.15
Monocyclic	Gamma-terpinene	99-85-4	C10H16	1062	1063	917	918	26.92	2.73
Monocyclic	Terpinolene	586-62-9	C10H16		1096	930	951	26.39	0.95
Monocyclic	Cis-p-menth-2-en-1-ol	29803-81-4	C10H18O	1099	1132	920	929	28.03	0.65
Monocyclic	Trans-p-menth-2-en-1-ol	29803-82-5	C10H18O		1152	903	913	24.99	0.38
Monocyclic	Terpinen-4-ol	562-74-3	C10H18O	1175	1193	916	917	55.07	7.28
Monocyclic	Terpineol	8000-41-7	C10H18O	1189	1207	911	917	38.8	3.11
Monocyclic	Cis-piperitol	16721-38-3	C10H18O	1253	1212	872	902	35.13	0.23
Monocyclic	Trans-piperitol	16721-39-4	C10H18O	1204	1225	896	929	68.3	0.34

Monocyclic	Alfa-terpinyl acetate	93836-50-1	C12H20O2		1335	913	924	72.44	0.11
Bicyclic	Sabinene	3387-41-5	C10H16	977	966	944	949	50.98	3.97
Bicyclic	Beta-thujene	28634-89-1	C10H16	930	1000	888	922	28.85	0.57
Bicyclic	Alfa-pinene	80-56-8	C10H16	939	1041	920	927	20.25	3.46
Bicyclic	5-isopropyl-2-methylbicyclo[3.1.0]hexan-2-ol#	546-79-2	C10H18O		1072	915	923	41.23	0.16
Bicyclic	Myrtenyl acetate	1079-01-2	C12H18O2		1343	895	903	47.83	0.11
Tricyclic	Bornyl acetate	76-49-3	C12H20O2	1289	1303	890	901	31.4	0.15
Tricyclic	Cis-3-acetoxy-1,8-cineole	81781-24-0	C12H20O3		1359	815	823	36.46	0.14
**Sesquiterpenes**									
Chain	(Z,E)-alfa -farnesene	26560-14-5	C15H24	1506	1863	870	889	26.61	0.13
Chain	Nerolidol	40716-66-3	C15H26O	1563	1917	922	947	56.53	1.05
Monocyclic	Beta-elemene	515-13-9	C15H24	1391	1739	913	918	25.1	1.01
Monocyclic	Gamma-elemene	29873-99-2	C15H24	1437	1784	910	923	25.68	0.3
Monocyclic	Humulene	6753-98-6	C15H24	1609	1805	899	905	33.04	0.41
Monocyclic	Hedycaryol	21657-90-9	C15H26O	1541	1904	910	920	28.39	0.19
Monocyclic	Germacrene B	15423-57-1	C15H24	1558	1912	911	918	20.35	0.19
Bicyclic	Caryophyllene	87-44-5	C15H24	1418	1769	945	947	32.4	0.19
Bicyclic	Bicyclogermacrene	24703-35-3	C15H24	1494	1851	911	918	22.09	0.81
Bicyclic	Gamma-amorphene	6980-46-7	C15H24		1869	907	914	15.56	0.4
Bicyclic	Cadina-1(10),4-diene	16729-01-4	C15H24		1879	913	929	43.81	1.02
Bicyclic	T-cadinol	5937-11-1	C15H26O	1462	1973	907	924	32.45	0.53
Bicyclic	Alfa-cadinol	481-34-5	C15H26O	1653	1981	899	905	43.46	0.7
Tricyclic	Spathulenol	77171-55-2	C15H24O	1578	1929	882	894	41.77	0.25
Tetracyclic	Cis-beta-copaene	18252-44-3	C15H24		1835	916	917	21.76	2.11

foods

Table S7 The influences of environmental factors on volatile oil profile in prickly ash pericarps

Name	Explains %	Contribution %	pseudo-F	P
ZA				
Pb	37.8	37.8	9.1	0.00**
MRH	9.7	9.7	2.6	0.07
pH	8.7	8.7	2.6	0.05*
MT	5.9	5.9	1.9	0.12
Long	7.2	7.2	2.6	0.08
K_t	4.6	4.6	1.8	0.16
N_a	6.0	6.0	2.7	0.03*
P_t	6.8	6.8	4.1	0.01**
As	3.8	3.8	4.2	0.01*
N_t	2.7	2.7	1.8	0.18
MAP	2.3	2.3	1.7	0.18
Cd	1.7	1.7	2.3	0.11
K_a	1.2	1.2	2.1	0.15
Al	0.8	0.8	1.7	0.25
P_a	0.5	0.5	1.2	0.42
Alt	0.4	0.4	<0.1	1.00
ZB1				
Long	10.6	13.9	3.2	0.04*
Mn	9.3	12.2	3.0	0.05*
Ni	4.7	6.1	1.5	0.20
Al	5.8	7.6	2.0	0.13
As	5.1	6.7	1.8	0.11
Cd	3.7	4.9	1.4	0.25
MAP	2.9	3.8	1.1	0.34
Alt	2.9	3.8	1.1	0.34
Ka	3.0	4.0	1.1	0.28
Pb	2.8	3.7	1.0	0.38
P_a	4.5	5.9	1.7	0.17
N_a	4.4	5.8	1.7	0.16
Lat	2.9	3.9	1.2	0.29
AtP	4.0	5.2	1.7	0.14
N_t	3.9	5.2	1.7	0.14
pH	1.5	2.0	0.6	0.61
MT	1.5	2.0	0.6	0.62
OM	1.0	1.4	0.4	0.79
P_t	0.9	1.1	0.3	0.84
K_t	0.5	0.6	0.2	0.97
MRH	0.3	0.3	<0.1	0.99
ZB2				

K_a	27.2	27.2	4.1	0.00**
AtP	18.7	18.7	3.5	0.03*
OM	17.5	17.5	4.3	0.03*
Pb	8.3	8.3	2.4	0.04*
Lat	7.3	7.3	2.5	0.07
N_a	5.7	5.7	2.2	0.11
Long	3.6	3.6	1.6	0.20
Mn	4.6	4.6	2.7	0.09
P_t	2.4	2.4	1.6	0.20
Al	2.1	2.1	1.8	0.28
K_t	2.0	2.0	4.9	0.19
Cd	0.4	0.4	<0.1	1.00

ZA (n= 17) represents green pericarps derived from *Zanthoxylum armatum*; ZB1 (n= 29) represents red pericarps derived from *Z. bungeanum* from Hancheng; ZB2 (n= 13) represents red pericarps from Fengxian; Lat represents latitude of the plantation; Long represents longitude of the plantation; Alt represents altitude of the plantation; AtP represents mean atmospheric pressure of the plantation; MAP represents mean annual precipitation of the plantation; MRH represents mean relative humidity of the plantation; MT represents mean temperature of the plantation; pH represents power of hydrogen in the soil; OM represents organic matter content in the soil; N_t represents the total nitrogen content in the soil; P_t represents the total phosphorus content in the soil; K_t represents the total potassium content in the soil; N_a represents available nitrogen content in the soil; P_a represents the available phosphorus content in the soil; K_a represents the available phosphorus content in the soil; Al represents aluminium content in the soil; As represents arsenic content in the soil; Cd represents cadmium content in the soil; Mn represents manganese content in the soil; Ni represents nickel content in the soil; Pb represents lead content in the soil.
